# Identification of *CLOCK* and *MARK2* as Candidate Blood-Based Biomarkers in Type 2 Diabetes: An Integrated Bulk and Single-Cell Transcriptomic Study

**DOI:** 10.3390/genes17101262

**Published:** 2026-10-11

**Authors:** Lili Yang, Jiahao Tang, Dongqing Zhang, Peng Yun

**Affiliations:** Department of Endocrinology, The Seventh Affiliated Hospital, Sun Yat-sen University, Shenzhen 518107, China

**Keywords:** T2DM, circadian *clock* genes, bioinformatics, scRNA-seq

## Abstract

**Background:** Type 2 diabetes mellitus (T2DM) is often accompanied by nervous system complications. However, the molecular mechanisms underlying T2DM remain incompletely understood. This study aimed to identify candidate blood-based biomarkers associated with T2DM by screening a neural-related gene set through integrated bulk and single-cell transcriptomic analysis of peripheral blood. The present analysis focused on the systemic expression patterns of the selected genes in peripheral blood as indicators of T2DM-associated immunometabolic dysregulation, rather than on neurological involvement. The neural-related gene set was used only as a predefined screening starting point. **Methods:** We employed a three-tier validation framework comprising (1) a training cohort (GSE15932, 8 T2DM patients vs. 8 controls) for biomarker discovery using differential expression analysis and machine learning (XGBoost, random forest, and PPI network topology); (2) an independent validation cohort (GSE184050, 50 T2DM patients vs. 65 controls) for cross-dataset diagnostic performance assessment (ROC analysis); and (3) an experimental validation cohort (5 T2DM patients vs. 5 controls) for independent clinical confirmation by RT-qPCR. Additionally, single-cell RNA sequencing (GSE268210) was used to investigate cell type-specific expression and immune regulatory networks. **Results:** By combining multiple screening methods and cross-dataset validation, we identified *CLOCK* and *MARK2* as key candidate genes from the neural-related gene set. *CLOCK* and *MARK2* showed high discriminatory performance in the discovery cohort, whereas their AUCs were 0.613 and 0.653, respectively, in the validation cohort, indicating limited standalone discriminatory ability. Both genes are highly expressed in peripheral blood monocytes and are associated with MIF-(CD74+CXCR4)-mediated cell communication, TNFA-NFκB inflammatory signaling, and activation of oxidative phosphorylation pathways. Finally, RT-qPCR in clinical samples confirmed that both genes were indeed upregulated (*p* < 0.05). **Conclusions:** *CLOCK* and *MARK2* were identified as candidate T2DM-associated blood-based biomarkers with reproducible expression differences across datasets. Their discriminatory performance was more modest in the independent validation cohort, indicating limited standalone diagnostic utility. Single-cell and computational analyses further suggested potential immunometabolic relevance. Because neurological phenotyping, cognitive assessment, and direct measures of nervous system involvement were not available in this study, any relationship between these genes and the neurological manifestations of T2DM remains speculative and hypothesis-generating, and requires prospective validation in cohorts with matched neurocognitive data.

## 1. Introduction

Type 2 diabetes mellitus (T2DM) is a complex metabolic disease driven by multiple factors such as genetics, obesity, and unhealthy lifestyle habits [1,2]. Chronic hyperglycemia, along with β-cell dysfunction, insulin resistance, and immune inflammation, intensifies the pathogenesis of T2DM [3]. To date, therapeutic strategies aimed at controlling blood glucose, improving insulin resistance, and delaying complications have made significant progress. However, there are still challenges, such as the limited long-term efficacy of medications, significant individual variability, and difficulties in fundamentally halting disease progression [4,5,6]. Determining the molecular pathological mechanisms of T2DM and identifying more precise and effective early diagnostic markers and therapeutic targets remain an urgent research priority.

The widespread impacts of T2DM on the nervous system are increasingly becoming a research focus. These impacts range from common peripheral neuropathy to more subtle forms of central impairment, such as cognitive decline [7,8]. People with T2DM appear to be more vulnerable to cognitive dysfunction, with multiple cognitive domains often affected [9,10]. This “peripheral-to-central” pattern of neural damage may stem from a complex set of underlying mechanisms.

Long-term hyperglycemia in T2DM can trigger chronic low-grade inflammation that may disrupt functional brain networks [11]. Chronic hyperglycemia in T2DM can trigger oxidative stress, accumulation of advanced glycation end products (AGEs), and abnormal tau phosphorylation, all of which may contribute to neuronal damage [12]. At the cellular level, impaired neuron–astrocyte metabolic coupling and neurovascular unit dysfunction have been proposed as links between metabolic disturbance and cognitive decline [13], and elevated plasma neurofilament light chain (NFL) correlates with cognitive deterioration in T2DM patients [14]. CNS dysfunction has also been suggested to contribute to systemic metabolic dysregulation rather than being merely a downstream complication [15]. Single-cell RNA sequencing (scRNA-seq) offers cellular-level resolution beyond bulk transcriptomics by resolving heterogeneous cell populations and their disease-associated expression states [16], making it suitable for localizing candidate genes to specific peripheral blood cell types.

Previous studies have employed transcriptomics, proteomics, and machine learning approaches to explore candidate biomarkers associated with T2DM [17,18], providing important evidence for understanding T2DM at the molecular level. However, differences in data sources, analytical strategies, and validation methods across studies mean that the reproducibility of candidate biomarkers in independent datasets still warrants further evaluation. Meanwhile, bulk RNA-seq reflects averaged expression signals across mixed-cell populations and lacks cellular-level resolution [19]; therefore, relying solely on bulk data makes it difficult to pinpoint the primary cellular sources and functional contexts of candidate genes. In addition, evaluating candidate genes derived from computational screening in independent cohorts and through experimental validation can further strengthen the supporting evidence. With these considerations in mind, integrating multi-method candidate gene screening, independent bulk transcriptome cohort evaluation, single-cell expression localization, and clinical sample experimental validation within a unified research framework would facilitate a systematic, multi-layered assessment of T2DM-related candidate genes.

Given the above research gaps, this study uses a predefined neural-related gene set only as a screening starting point, with an emphasis on evaluating the disease-associated expression characteristics and cellular contexts of the selected genes in peripheral blood from T2DM patients. Specifically, we first applied a combination of differential expression analysis, XGBoost, random forest, and protein–protein interaction (PPI) network analysis in the GSE15932 training cohort to screen for candidate key genes. We then evaluated the reproducibility of expression changes and the discriminatory ability between T2DM patients and controls for these candidate genes in the independent GSE184050 cohort. Furthermore, we utilize peripheral blood single-cell RNA sequencing data to characterize the cell type distribution of candidate genes and to explore associated cell–cell communication and inflammatory and metabolic states. Finally, we perform RT-qPCR validation using independent clinical blood samples. Through this analytical framework, this study aims to identify T2DM-related candidate genes with cross-cohort expression support and preliminary experimental evidence while further providing cellular-level insights into their peripheral immunometabolic contexts. The central aim of this study is to identify and validate blood-based candidate biomarkers for T2DM, using a neural-related gene set as the screening starting point.

## 2. Materials and Methods

### 2.1. Data Source

The GSE15932 dataset, sourced from the Gene Expression Omnibus (GEO, https://www.ncbi.nlm.nih.gov/gds (accessed on 25 September 2025)), served as the training cohort (discovery set). Its modest size (8 per group) was offset by reserving GSE184050 (*n* = 115) as an independent validation set to prevent data leakage and ensure generalizability. Multi-algorithm consensus screening was used to minimize overfitting. Differential expression analysis, machine learning-based feature selection, PPI network analysis, and diagnostic model (nomogram) construction were performed on this dataset. The GSE184050 dataset, serving as the independent validation set (platform: GPL11154), contains blood samples from 50 T2DM patients and 65 healthy controls. This dataset was used exclusively to validate the expression patterns and consistency of the candidate key genes identified from the training set. The GSE268210 single-cell dataset comprises blood samples from 10 T2DM patients (platform: GPL16791) and contains no healthy control donors; this was used for cellular-level mechanistic exploration. For experimental validation via RT-qPCR confirmation, an independent clinical cohort of 5 T2DM patients and 5 healthy controls was recruited. Additionally, 1889 neural-related genes (NRGs) were sourced from a published study [20] (Table S1). The sample composition, data type, analytical role, and availability of demographic and clinical information for each cohort are summarized in Table 1.

Detailed demographic and clinical variables, including age, sex, neurological status, cognitive function, and the presence of diabetes-related complications, were not consistently available for the public datasets used in this study. Therefore, subgroup analyses or covariate-adjusted analyses according to these clinical characteristics could not be performed. The GSE268210 single-cell cohort only contains patients with T2DM and does not include healthy controls or neurological phenotyping.

GSE15932 was selected as the training cohort because it meets the predefined criteria for T2DM status, availability of healthy controls, and blood-derived transcriptomic profiling. Although its sample size is modest (8 samples per group), it was used as an exploratory discovery cohort for initial differential expression analysis and feature screening rather than as the sole dataset supporting the study findings. The substantially larger GSE184050 cohort was reserved exclusively for independent validation to avoid information leakage and to assess the reproducibility of the identified candidate key genes across datasets. Multiple feature selection algorithms were used to identify consistently selected candidate genes; nevertheless, the limited sample size of the discovery cohort remains a methodological limitation.

### 2.2. Identification of Neural-Related Hub Genes in T2DM

The limma package (v 3.54.0) [21] was applied to the GSE15932 training set to detect DEGs between the T2DM and control samples (log_2_FC > 0.5, p.adj < 0.05). The DEGs were visualized in volcano plots using ggplot2 (v 3.5.2) [22] and in heatmaps using ComplexHeatmap (v 2.14.0) [23]. Determination of the overlap between the DEGs and NRGs via ggvenn (v 0.1.9) [24] produced a set of candidate genes (CGs) for subsequent analysis. We then performed GO (BP, MF, and CC) and KEGG enrichment analyses on these CGs using clusterProfiler (v 4.10.1) [25], with a significance cutoff of *p* < 0.05. The top 5 GO terms and top 10 KEGG pathways are reported. A PPI network for the CGs was built using the STRING database (https://string-db.org/) with an interaction score ≥ 0.15 for *Homo sapiens* and visualized with circsize (v 0.4.15) [26]. This permissive threshold (0.15, the lowest pre-computed STRING threshold) was used to retain sufficient edges for topology-based hub ranking in a small candidate gene set. The final key genes were not selected based on this threshold alone but based on the intersection of three independent ranking methods (XGBoost, random forest, and CytoHubba centrality). Hub gene selection was performed using three CytoHubba algorithms (DMNC, Clustering Coefficient, and Closeness), with HGs defined as the intersection of the top 20 genes from each ranking.

### 2.3. Identification and Dual-Cohort Validation of Key Genes (KGs)

To ensure robustness and minimize overfitting, candidate key genes were subjected to dual-cohort validation (training and independent validation sets) prior to experimental confirmation. To prioritize candidate genes, we applied XGBoost (v 1.7.3.1) to the CGs using the training set samples, with hyperparameters configured as nround = 200, eta = 0.3, and max_depth = 6 [27]. The five genes exhibiting the greatest feature importance were retained as XGBoost-derived feature genes (XFGs). Separately, a random forest classifier was built for the CGs via the randomForest package (v 4.1.4) [28], and the ntree parameter was optimized by evaluating values from 10 to 100 in increments of 2, selecting the one that yielded the minimum out-of-bag (OOB) error. Features with an importance score exceeding zero were extracted as random forest feature genes (RFGs). The overlap between the XFG and RFG sets was then computed using ggvenn (v 0.1.9) to obtain candidate key genes (CKGs). We next assessed the expression levels of these CKGs in both the training and validation cohorts using the Wilcoxon rank-sum test. Genes showing statistically significant differences (*p* < 0.05) and consistent directional changes across both datasets were designated as key genes (KGs). Given the small size of the GSE15932 discovery cohort (8 T2DM patients vs. 8 controls), formal stability selection such as repeated cross-validation or bootstrap resampling was not performed. Instead, candidate genes were required to be supported by multiple independent screening strategies (XGBoost, random forest, and PPI network topology) and to show consistent expression changes in the independent GSE184050 cohort. These consensus and cross-cohort criteria are pragmatic robustness checks rather than formal stability analyses, and the risk of overfitting in the discovery cohort cannot be fully excluded.

### 2.4. ROC Analysis, Correlation Analysis, and Genemania Analysis of KGs

To evaluate the discriminatory ability of the KGs, receiver operating characteristic (ROC) curve analyses were performed separately in the GSE15932 discovery/training cohort and the GSE184050 validation cohort using the pROC package (v 1.18.5) [29]. *CLOCK* and *MARK2*, identified from the discovery workflow, were evaluated using their normalized expression values as individual predictors of T2DM status. The area under the ROC curve (AUC) and corresponding 95% confidence interval (CI) were calculated for each gene in each cohort. In the validation cohort, no additional machine learning feature selection was performed before ROC evaluation. Notably, these analyses assessed the cross-dataset discriminatory performance of the individual genes rather than externally validating the combined two-gene nomogram, which was constructed only for the GSE15932 cohort.

### 2.5. Gene Set Enrichment Analysis (GSEA)

The KEGG reference gene set (c2.cp.kegg.v7.5.1.symbols.gmt) was downloaded from the Molecular Signatures Database (MSigDB; https://www.gsea-msigdb.org/gsea/msigdb (accessed on 25 September 2025)). Using the GSE15932 cohort, we calculated the Spearman correlation coefficients between the KGs and all remaining genes via the psych package (v 2.2.9), ranking genes by their correlation values in descending order. To explore T2DM-related signaling pathways associated with the KGs, we performed GSEA using clusterProfiler (v 4.7.1), with significance thresholds set at *p* < 0.05 and |normalized enrichment score (NES)| > 1. The top 5 pathways, ranked by |NES|, are reported. The overlapping pathways among the KGs were subsequently identified using ggvenn (v 0.1.9).

### 2.6. Single-Cell RNA Sequencing (scRNA-Seq) Data Analysis

We analyzed the single-cell RNA-seq dataset GSE268210 using the Seurat pipeline (v 5.0.1) [30]. Following the creation of Seurat objects, we applied quality control filters of 200 < nFeatures < 3000, nCount_RNA < 10,000, and percent.mt < 5%, with the distributions of these metrics visualized before and after filtering. After data normalization, we identified the top 2000 HVGs via the VST method implemented in FindVariableFeatures, labeling the top 10 with LabelPoints. The ScaleData function was then applied for data scaling, followed by PCA on the HVGs using RunPCA. To select the optimal PC number, we performed the Jackstraw permutation test (PCs 1–50) and examined the variance decay pattern via ElbowPlot, retaining PCs meeting both *p* < 0.05 and a plateau position in the elbow plot (the first 20 PCs). Cell clustering was then performed using FindNeighbors and FindClusters (resolution = 0.1), and the results were visualized with t-SNE. Marker genes for each cluster were identified using FindAllMarkers (min.pct = 0.25, only.pos = TRUE, logfc.threshold = 1), and cell types were annotated by cross-referencing these markers with literature-reported markers [31] and the CellMarker database (http://xteam.xbio.top/CellMarker/ (accessed on 25 September 2025)). The annotated clusters were visualized with t-SNE, and bubble plots generated via DotPlot were used to illustrate the marker gene expression profiles. Finally, we examined KG expression across cell types and designated cells with both literature evidence and high KG expression as key cells.

To evaluate inter-donor variability in *CLOCK* and *MARK2* expression, an additional donor-stratified analysis was performed across the 10 T2DM samples in GSE268210. For each donor, the mean and median normalized expression levels, standard deviations, numbers of gene-expressing cells, total cell numbers, and proportions of cells with non-zero *CLOCK* or *MARK2* expression were calculated. Donor-wise expression distributions, mean expression levels, and proportions of gene-expressing cells were visualized separately. This analysis was used to assess whether the overall single-cell expression patterns of *CLOCK* and *MARK2* were disproportionately driven by individual donors and to characterize inter-individual heterogeneity. Because GSE268210 includes only T2DM donors and no healthy controls, this donor-stratified analysis was not used to infer disease-specific differential expression.

### 2.7. Cell Communication, Pseudotime, Functional Enrichment, and Metabolic Pathway Analyses

To explore intercellular crosstalk between key cells and other populations, we applied CellChat (v 1.6.1) [32] to the GSE268210 dataset for cell–cell communication analysis. Via this approach, potential ligand–receptor pairs were quantified, yielding interaction counts and strengths, along with bubble plots visualizing these interactions. To reconstruct the developmental trajectory of key cells and track KG expression dynamics, we performed pseudotime analysis on the key cell subsets. Specifically, we re-clustered the dimension-reduced key cell populations using clusterCells (resolution = 0.4) and constructed single-cell trajectories with monocle (v 2.26.0) [33], simultaneously plotting KG expression trends along the pseudotime axis. For functional characterization of cell subpopulations, we employed irGSEA (v 3.3.2) [34], applying its six complementary algorithms (AUCell, UCell, singscore, ssGSEA, JASMINE, and Viper) via the irGSEA.score function to score Hallmark and KEGG gene sets from MSigDB. Pathways with differential activity across subpopulations were identified using the Wilcoxon rank-sum test (*p* < 0.05). To consolidate results across algorithms, we applied the rank aggregation method implemented in RobustRankAggreg (v 1.2.1) [35] to integrate the six sets of enrichment outcomes, retaining only pathways that achieved significance in the majority of algorithms (*p* < 0.05). Finally, we used the VISION algorithm within scMetabolism (v 0.2.1) [36] to score metabolic pathway activity per cell, enabling comparison of metabolic states across distinct cell types.

### 2.8. Reverse Transcription Quantitative Polymerase Chain Reaction (RT-qPCR)

To investigate KG expression levels in clinical samples, 5 blood samples from T2DM patients and 5 from control subjects were acquired during routine outpatient clinic hours (approximately 08:00–10:00) at The Seventh Affiliated Hospital, Sun Yat-sen University. This RT-qPCR cohort served as the third tier of our evidence framework, following bioinformatics discovery and independent validation. The small size (5 per group) of this cohort reflects the exploratory nature of this experimental layer, which aimed to verify computational predictions in prospectively collected specimens rather than establish population-level diagnostic thresholds. Detailed demographic and clinical variables, including age, sex, diabetes duration, HbA1c, BMI, medication use, and the presence of diabetes-related complications, were not systematically recorded for this cohort. Therefore, subgroup analyses or covariate-adjusted analyses according to these clinical characteristics could not be performed. All participants provided informed consent by signing and completing a form. Ethical approval was granted by the Medical Ethics Committee of the Seventh Affiliated Hospital of Sun Yat-sen University (Shenzhen), under Ethics Approval Number KY-2025-413-02. Data were plotted and *p*-values computed using GraphPad Prism 10. Total RNA was isolated from the ten enrolled samples via TRIzol reagent (Vazyme Biotech Co., Ltd., Nanjing, China) and subsequently reverse-transcribed to cDNA using an HP All-in-One qRT Master Mix II RT203-Ver.1 kit (Yungen Biological Technology Co., Ltd., Kunming, China). RT-qPCR was then run with the 2 × Universal Blue SYBR Green RT-qPCR Master Mix (Yungen Biological Technology Co., Ltd., Kunming, China), and GAPDH was used as the reference gene for data normalization. The primers applied in RT-qPCR are provided in Table S2. Gene expression fold changes between the T2DM and control groups were determined by the 2^−ΔΔCt^ method, with statistical significance assessed by the *t*-test at a threshold of *p* < 0.05. Given the small sample size (*n* = 5 per group), normality could not be reliably assessed, and the *t*-test should therefore be regarded as an exploratory test; the corresponding results should be interpreted with caution. A non-parametric test would be more appropriate in larger cohorts, and future studies with adequately powered sample sizes should either formally assess distributional assumptions or use non-parametric methods.

### 2.9. Supplementary Analyses

Exploratory analyses, including nomogram construction and decision curve analysis (Supplementary Methods S1), subcellular and chromosomal localization (Supplementary Methods S2, [37]), immune cell infiltration using xCell (https://github.com/dviraran/xCell (accessed on 25 September 2025)) (Supplementary Methods S3, [38]), drug prediction and molecular docking (Supplementary Methods S4), and molecular dynamics simulations (Supplementary Methods S5), were performed as supporting analyses. Detailed protocols are provided in the Supplementary Methods. These analyses were not used to support the primary conclusions of the study.

### 2.10. Statistical Analysis

R software (v 4.2.3) was used for all analyses. In addition, the Wilcoxon test was employed to assess group disparities, with the significance threshold set at *p* < 0.05. Multiple-testing correction was not applied in the exploratory enrichment, immune cell infiltration, or single-cell pathway analyses; *p*-values are therefore reported as nominal values, and these results should be interpreted as hypothesis-generating only. Differential expression analysis used Benjamini–Hochberg-adjusted *p*-values (limma default).

## 3. Results

### 3.1. Multi-Algorithm Screening and Dual-Cohort Validation Identify CLOCK and MARK2 as Candidate T2DM-Associated Genes

**Figure 1:**
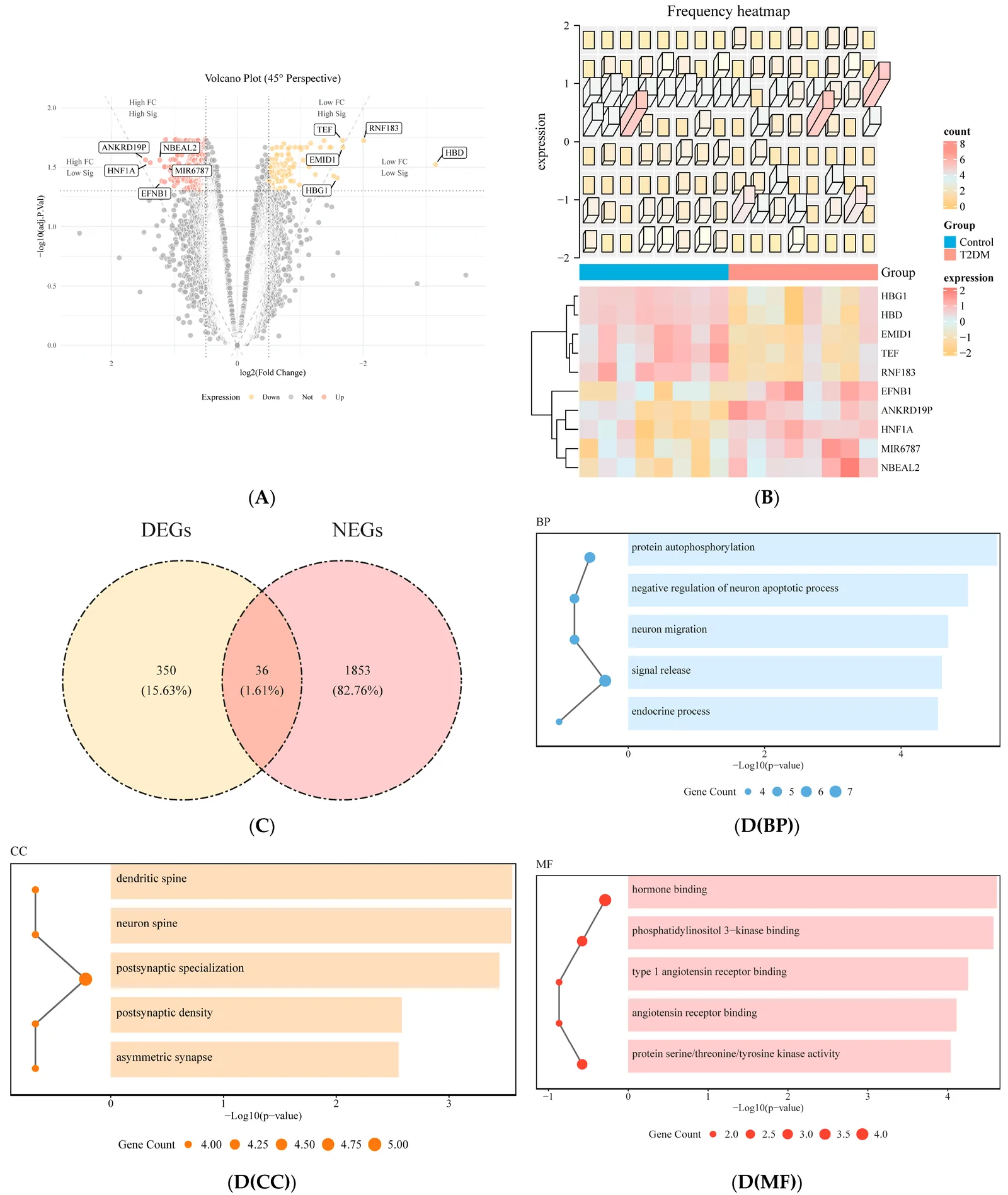

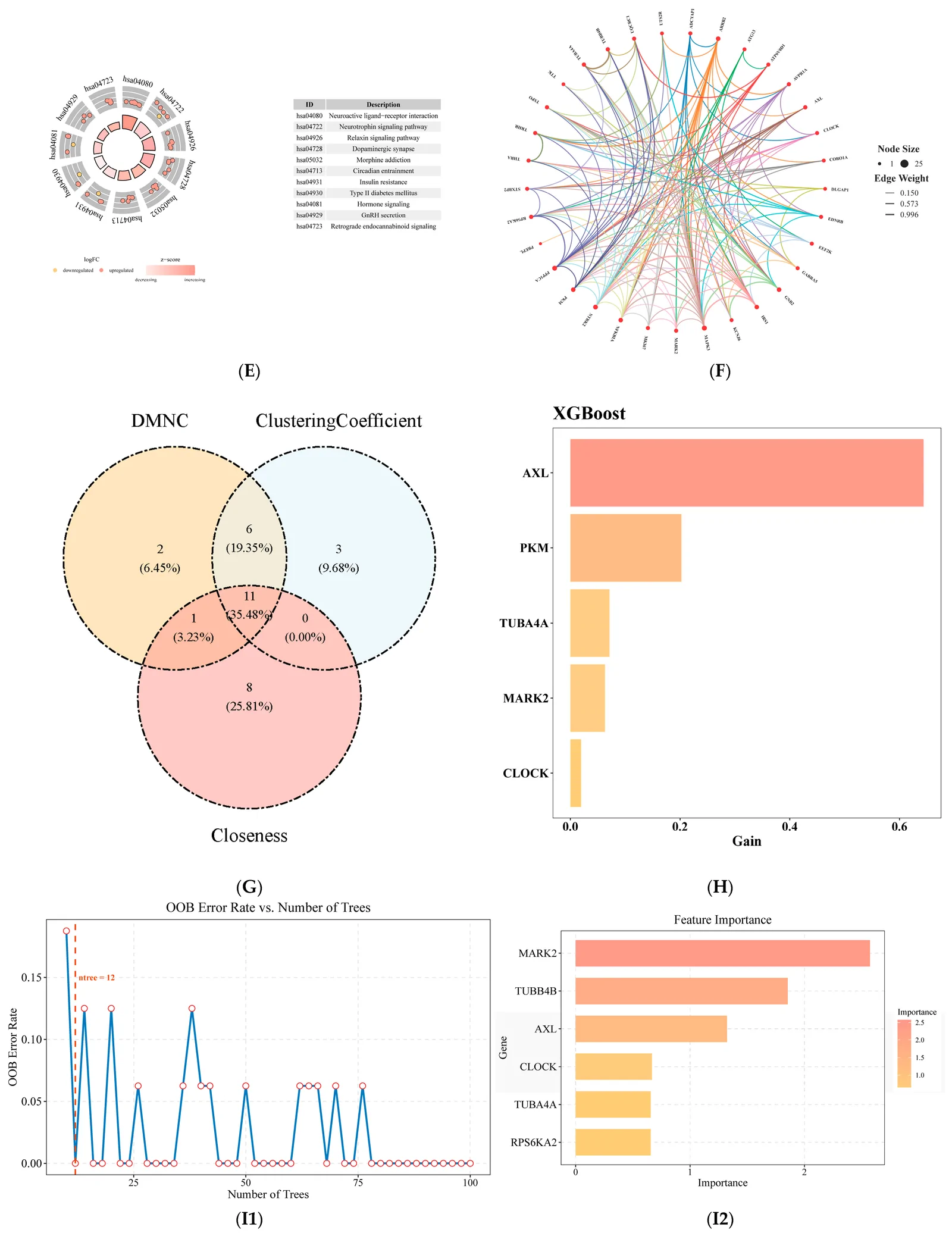

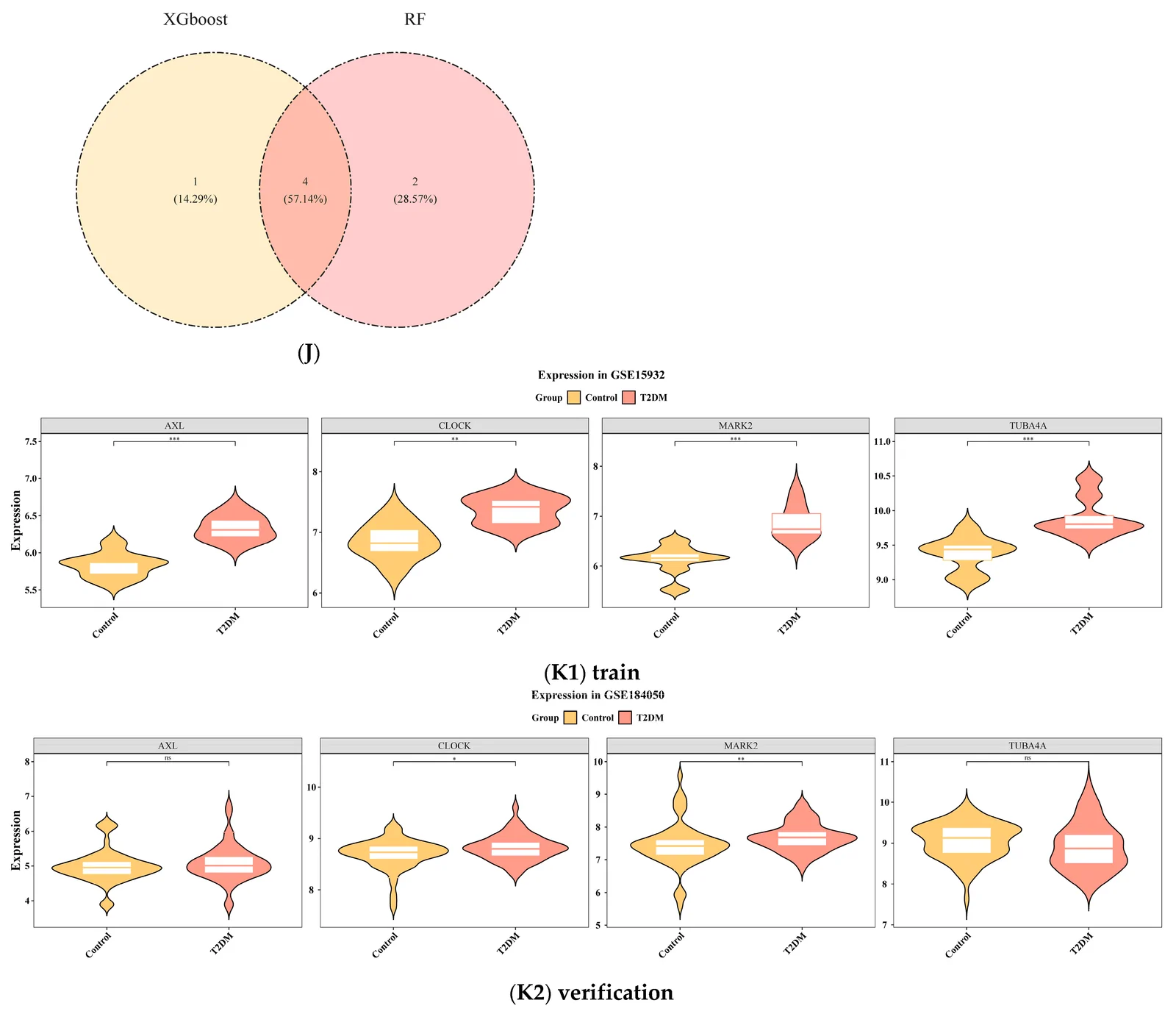
Multi-algorithm screening identified *CLOCK* and *MARK2* as candidate T2DM-associated genes. (**A**) Volcano plot of differentially expressed genes (DEGs; |log_2_FC| > 0.5, adjusted *p* < 0.05) in the GSE15932 training cohort (8 T2DM patients vs. 8 controls). Top 5 upregulated and downregulated genes are labeled. (**B**) Heatmap of the top 20 DEGs across all 16 samples. (**C**) Venn diagram of DEGs and 1889 neural-related genes (NRGs), yielding 36 candidate genes (CGs). (**D**) Top 5 significantly enriched Gene Ontology (GO) terms for biological process (BP), cellular component (CC), and molecular function (MF) among the 36 CGs, analyzed using clusterProfiler (*p* < 0.05). (**E**) Top 10 significantly enriched Kyoto Encyclopedia of Genes and Genomes (KEGG) pathways among the CGs, sorted by nominal *p*-value without multiple-testing correction. (**F**) Protein–protein interaction (PPI) network of 36 CGs (STRING, interaction score ≥ 0.15). This permissive threshold was used because the candidate gene set was small; a higher threshold would have removed too many edges and limited topology-based hub ranking. (**G**) Intersection of top 20 genes ranked by three CytoHubba algorithms (DMNC [double-specificity maximum neighborhood component], Clustering Coefficient, and Closeness), yielding 11 hub genes. (**H**) Top 5 XGBoost feature genes. (**I1**,**I2**) Random forest (RF) feature genes with importance > 0. (**J**) Intersection of XGBoost and RF features, yielding 4 candidate key genes: *MARK2*, *AXL*, *CLOCK*, and *TUBA4A*. (**K1**,**K2**) Expression of the 4 candidates in the training (GSE15932; 8 T2DM patients vs. 8 controls) and independent validation cohorts (GSE184050; 50 T2DM patients vs. 65 controls). Violin plots show normalized expression distributions, with the horizontal line indicating the median. *CLOCK* and *MARK2* were consistently upregulated in T2DM (Wilcoxon test, nominal *p* < 0.05 without multiple-testing correction) and designated as key genes. Significance: * (*p* < 0.05), ** (*p* < 0.01), *** (*p* < 0.001), ns (not significant).

Our study design followed a three-tier validation framework: (i) discovery and multi-algorithm screening in the GSE15932 training cohort (*n* = 16); (ii) diagnostic performance validation in the independent GSE184050 cohort (*n* = 115); and (iii) experimental confirmation by RT-qPCR in an independent clinical cohort (*n* = 10). It should be noted that, although the training cohort (GSE15932) is modest in size, the subsequent validation in the substantially larger independent GSE184050 cohort (*n* = 115) and the use of multi-algorithm consensus screening partially mitigate concerns regarding overfitting and dataset-specific findings. However, formal stability selection was not performed, and overfitting cannot be fully excluded. Differential expression analysis was conducted on the training set samples, leading to the identification of 386 DEGs in the T2DM samples, including 273 upregulated genes and 113 downregulated genes (Table S3). Compared with the control group, ANKRD19P and HNF1A were markedly elevated in the T2DM group, whereas RNF183 and HBD were downregulated. These genes represent differentially expressed candidates identified from the cross-sectional discovery cohort; however, this design precludes causal inference, and their altered expression may reflect consequences of the disease state rather than causal drivers of T2DM pathogenesis (Figure 1A,B). To further focus on genes with a neural-related annotation in T2DM, the intersection of the DEGs and NRGs was calculated, resulting in the screening of 36 CGs, including EEF2K and NTRK2 (Figure 1C and Table S4). Functional enrichment analysis was subsequently conducted on the CGs. GO analysis identified 739 significant terms (*p* < 0.05), among which 605 were BPs involving pathways such as protein autophosphorylation and negative regulation of neuronal apoptotic process; 48 were CCs, including dendritic spine and neuron spine; and 86 were MFs, such as hormone binding and phosphatidylinositol 3-kinase binding (Figure 1D(BP,CC,MF) and Table S5). KEGG analysis yielded 70 significant pathways (*p* < 0.05) (Table S6), including the neuroactive ligand–receptor interaction and neurotrophin signaling pathway (Figure 1E). In addition, PPI network analysis highlighted close functional couplings among 33 of the CGs, indicating that they may occupy core positions in the T2DM-related molecular regulatory network (Figure 1F). Determination of the intersection of the top 20 genes screened by the three algorithms resulted in 11 HGs, which may synergistically regulate the key molecular network involved in neural function and metabolic homeostasis in T2DM (Figure 1G and Table S7).

To screen for robust KGs, first, XGBoost analysis was performed, leading to the identification of five XFGs, and RF analysis was conducted to select six RFGs (Figure 1H,I1,I2). By examining the intersection of these two sets of feature genes, four common CKGs were identified: *MARK2*, *AXL*, *CLOCK*, and *TUBA4A* (Figure 1J). Next, the expression levels of the CKGs were assessed for validation in both the training and validation sets. In both datasets, *CLOCK* and *MARK2* were significantly upregulated in the T2DM group compared to the control group, showing clear differences between the two groups (Figure 1K2,K2). Therefore, these genes were identified as the KGs in T2DM.

### 3.2. ROC Evaluation of CLOCK and MARK2 Across the Discovery and Validation Cohorts

**Figure 2:**
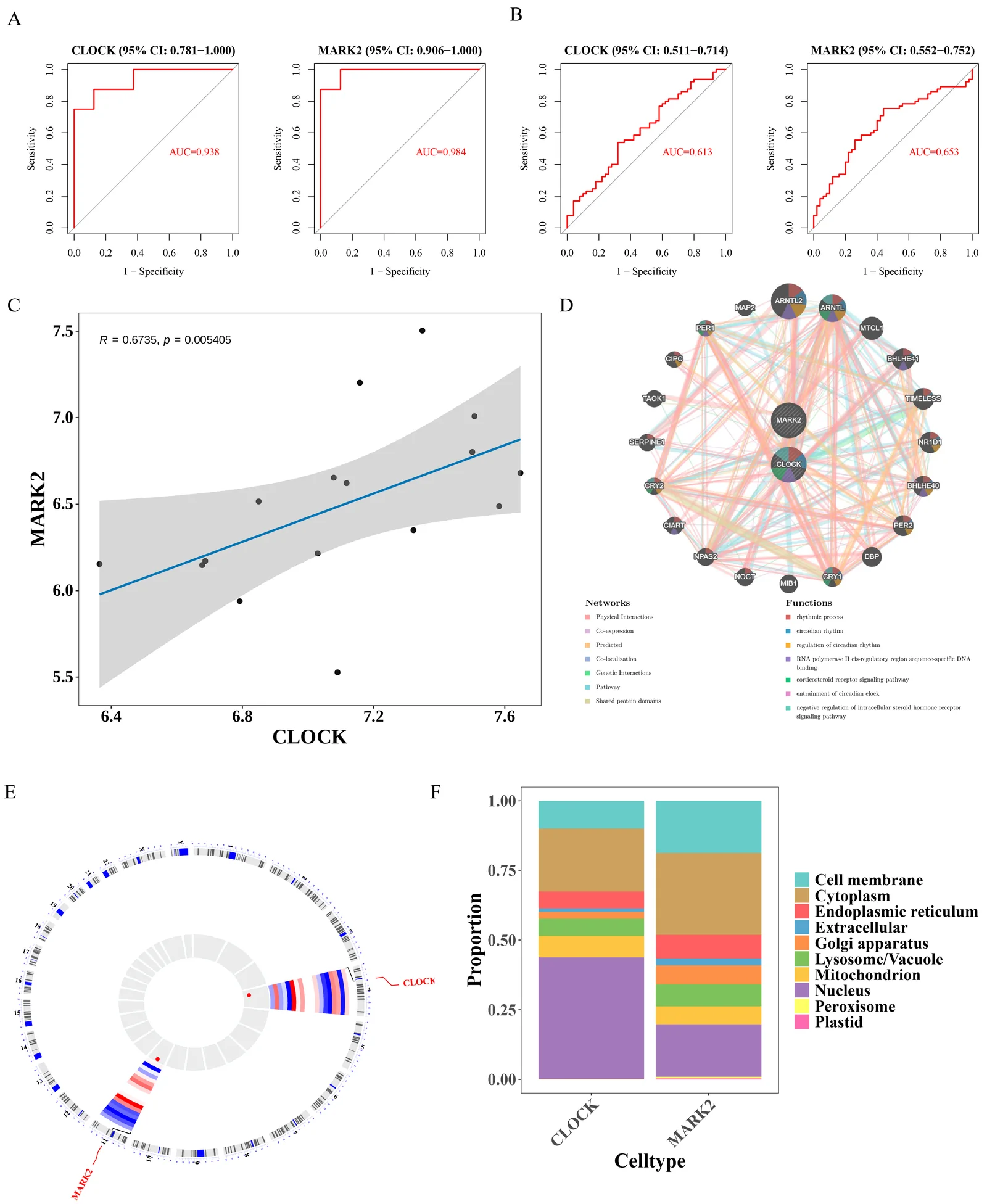
Comprehensive characterization of KGs. (**A**) Receiver operating characteristic (ROC) curves for *CLOCK* and *MARK2* in the training cohort (AUC > 0.9). (**B**) ROC curves of *CLOCK* and *MARK2* in the GSE184050 validation cohort. The corresponding area under the curve (AUC) values and 95% confidence intervals (CIs) are shown. (**C**) Spearman correlation between *CLOCK* and *MARK2* (r = 0.67, *p* = 0.005). (**D**) GeneMANIA interaction network showing 20 related genes enriched in rhythmic process and circadian *clock* regulation. (**E**) Chromosomal localization of *CLOCK* (chromosome 4) and *MARK2* (chromosome 11). (**F**) Subcellular localization predicted by DeepLoc-2.0. Sample sizes: GSE15932 discovery cohort, 8 T2DM patients vs. 8 controls; GSE184050 validation cohort, 50 T2DM patients vs. 65 controls. ROC curves were generated using the pROC package, and AUC with 95% CI is shown for each gene. Spearman correlation in (**C**) was based on the GSE15932 cohort. GeneMANIA network in (**D**) shows 20 genes functionally associated with *CLOCK* and *MARK2*. Subcellular localization in (**F**) was predicted using DeepLoc-2.0. Abbreviations: ROC, receiver operating characteristic; AUC, area under the curve; CI, confidence interval.

In the GSE15932 discovery/training cohort, *CLOCK* and *MARK2* showed high discriminatory performance, with AUCs of 0.938 (95% CI: 0.781–1.000) and 0.984 (95% CI: 0.906–1.000), respectively (Figure 2A). When the same two genes were evaluated in the GSE184050 validation cohort, their discriminatory performance was attenuated, with AUCs of 0.613 (95% CI: 0.511–0.714) for *CLOCK* and 0.653 (95% CI: 0.552–0.752) for MARK2 (Figure 2B). Spearman correlation analysis further revealed a significant positive correlation between the expression levels of *MARK2* and *CLOCK* (r = 0.67, *p* = 0.005) (Figure 2C). The interaction network constructed via GeneMANIA (https://genemania.org/) showed 20 genes closely associated with *CLOCK* and *MARK2*. The enriched biological terms for these genes included rhythmic process, circadian *clock* regulation, RNA polymerase II cis-regulatory region binding, and corticosteroid receptor signaling (Figure 2D). Mapping of chromosomal positions revealed that *CLOCK* resided on chromosome 4, whereas *MARK2* was located on chromosome 11 (Figure 2E). Subcellular localization prediction suggested that the CLOCK protein was mainly distributed in the nucleus, followed by the cytoplasm, while the MARK2 protein was primarily localized in the cytoplasm, with additional distribution in the cell membrane and nucleus (Figure 2F). Collectively, CLOCK and MARK2 showed only modest individual discriminatory performance in the larger independent validation cohort (AUC 0.613 and 0.653, respectively), despite their consistent expression differences. Their co-expression and network associations further provide exploratory clues regarding their potential involvement in circadian-related molecular processes.

### 3.3. KGs Co-Regulate Core Pathways of T2DM and Are Associated with Immune Microenvironment Characteristics

**Figure 3:**
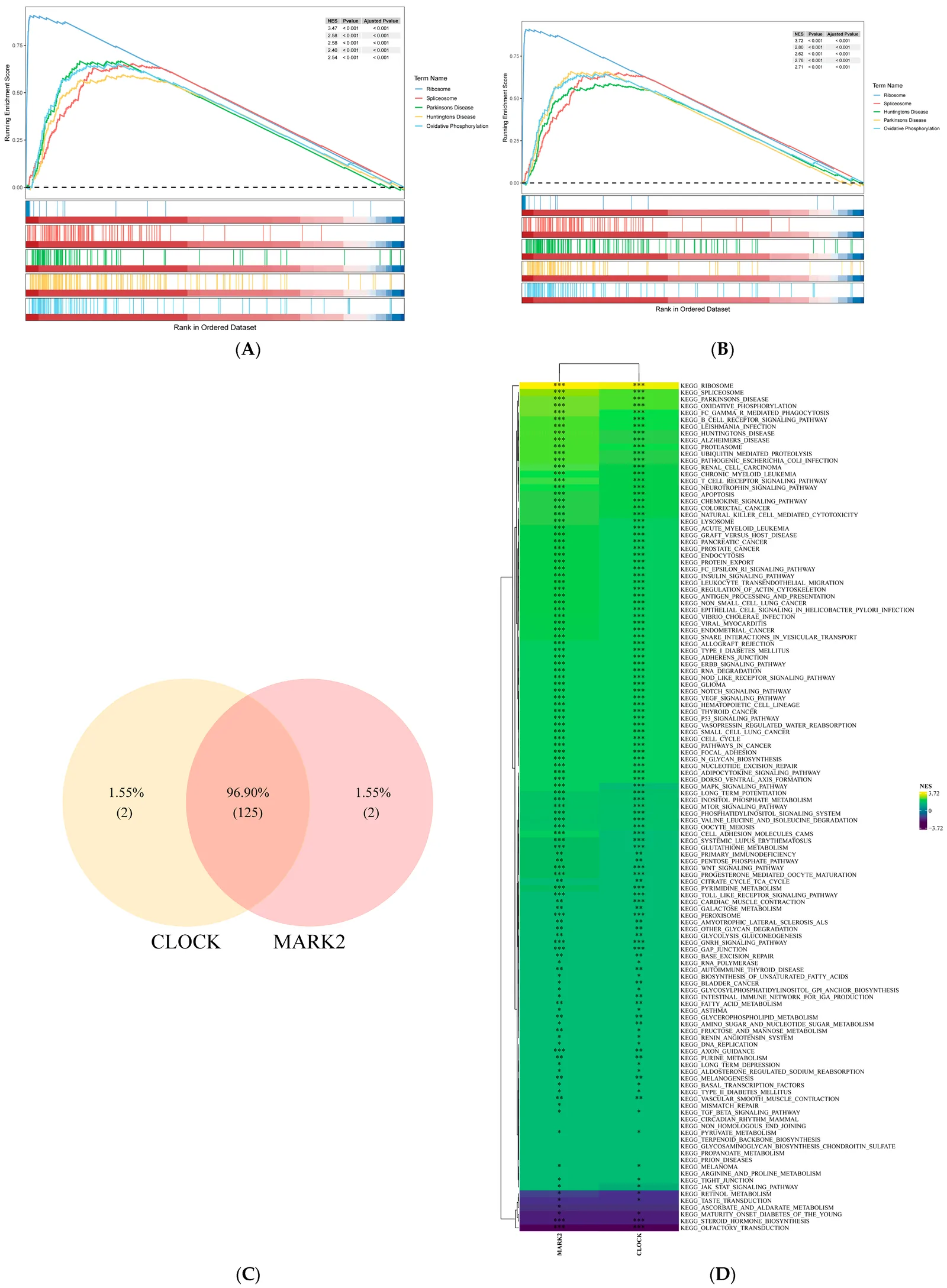

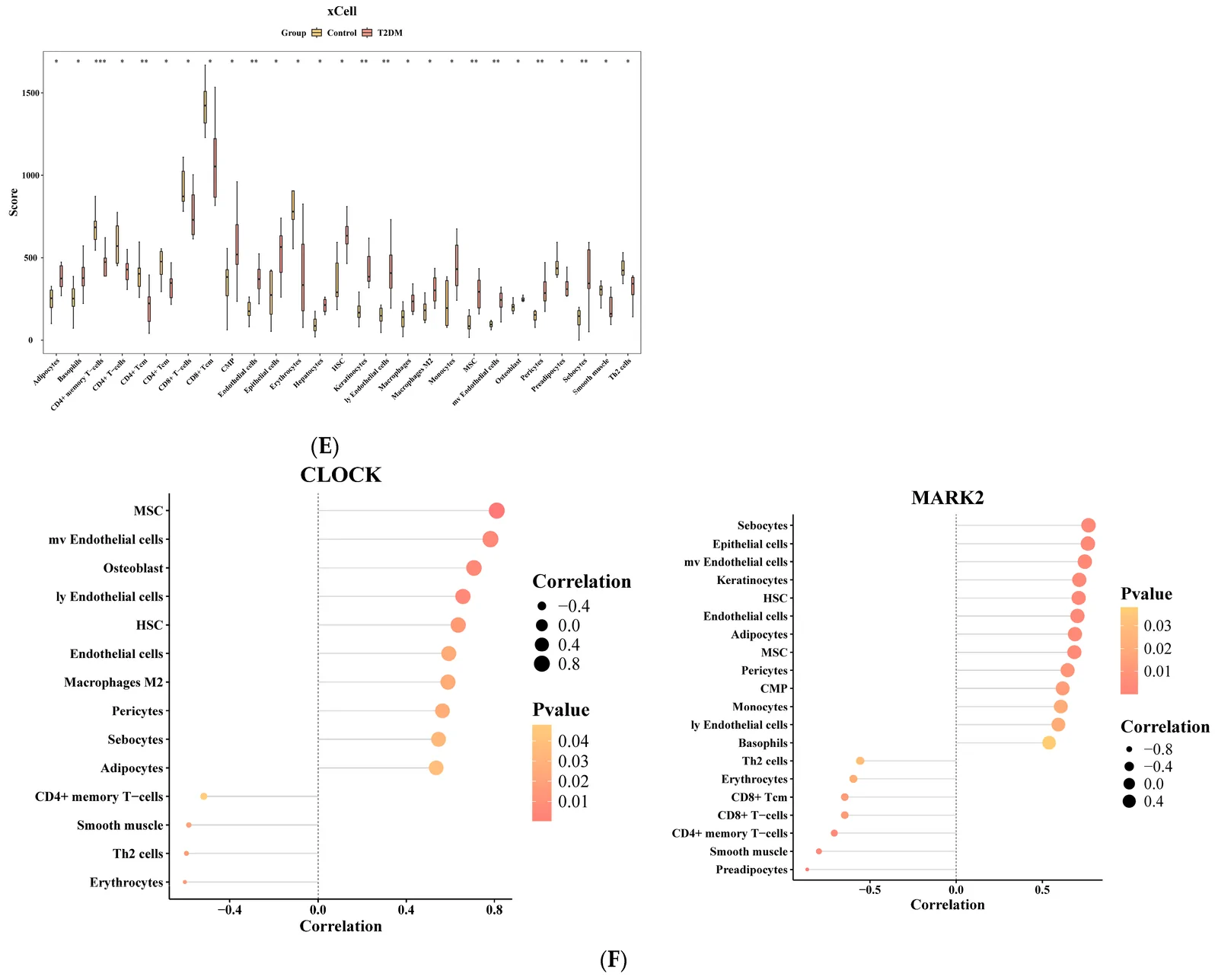
KGs co-regulate core pathways of T2DM and are associated with immune microenvironment characteristics. (**A**,**B**) Gene set enrichment analysis (GSEA) showing the top 5 enriched KEGG pathways for *CLOCK* (**A**) and *MARK2* (**B**) in the GSE15932 cohort, ranked by |normalized enrichment score (NES)| (*p* < 0.05, |NES| > 1). (**C**) Venn diagram showing 125 common enriched pathways shared by *CLOCK* and *MARK2*. (**D**) Heatmap of GSEA enrichment scores for the top shared pathways, including KEGG_TYPE_II_DIABETES_MELLITUS, KEGG_INSULIN_SIGNALING_PATHWAY, KEGG_GLYCOLYSIS_GLUCONEOGENESIS, and KEGG_NF_KAPPA_B_SIGNALING_PATHWAY. (**E**) Exploratory xCell deconvolution of the GSE15932 cohort (8 T2DM patients vs. 8 controls), showing inferred enrichment scores for cell types that differed between groups (Wilcoxon rank-sum test, nominal *p* < 0.05, without multiple-testing correction). These estimates are based on only 16 samples and are presented as exploratory. (**F**) Correlation heatmap between DEICs and key genes (*CLOCK* and *MARK2*). *CLOCK* showed a strong positive correlation with mesenchymal stem cells (MSCs) and a negative correlation with erythrocytes. *MARK2* showed a strong positive correlation with sebocytes and a negative correlation with preadipocytes. Sample size: GSE15932 cohort, 8 T2DM patients vs. 8 controls. GSEA was performed using clusterProfiler, and pathways with *p* < 0.05 and |NES| > 1 are shown. The xCell analysis in (**E**) was exploratory; *p*-values are nominal and were not corrected for multiple testing because of the small sample size (*n* = 16). The correlation analysis in (**F**) was based on inferred cell type enrichment scores. Abbreviations: GSEA, gene set enrichment analysis; NES, normalized enrichment score; KEGG, Kyoto Encyclopedia of Genes and Genomes; DEICs, differentially expressed immune cells; MSCs, mesenchymal stem cells. Significance: * (*p* < 0.05), ** (*p* < 0.01), *** (*p* < 0.001).

The functional enrichment analysis results showed that *CLOCK* and *MARK2* were enriched in 127 pathways each, including KEGG_RIBOSOME and KEGG_SPLICEOSOME (Figure 3A,B; Tables S8 and S9). Notably, there were 125 common enriched pathways between the two genes (Figure 3C). Among these, pathways closely related to the pathological process of T2DM were identified, such as KEGG_TYPE_II_DIABETES_MELLITUS, KEGG_INSULIN_SIGNALING_PATHWAY, KEGG_GLYCOLYSIS_GLUCONEOGENESIS, and KEGG_NF_KAPPA_B_SIGNALING_PATHWAY.

The heatmap indicated that both KGs were significantly enriched in the KEGG_RIBOSOME pathway (Figure 3D), suggesting that the KGs may affect T2DM-related pathophysiological processes by regulating basic cellular processes such as ribosome biogenesis or protein synthesis. Exploratory xCell-based deconvolution of the GSE15932 cohort suggested differences in inferred enrichment scores for several cell types between T2DM and control samples (Figure 3E and Figure S1). However, because this analysis was based on only 16 samples and because *p*-values are reported as nominal values without multiple-testing correction, these estimates are unstable and are presented here as exploratory only. Correlation analysis between *CLOCK*/*MARK2* expression and inferred cell type enrichment scores suggested several association signals, including positive associations of *CLOCK* with inferred MSC scores and of *MARK2* with inferred sebocyte scores, and negative associations with inferred erythrocyte and preadipocyte scores (Figure 3F; Table S10). These cell types are not expected to be abundant in peripheral blood; their inferred enrichment most likely reflects shared transcriptomic features with circulating cells and the limited tissue specificity of the xCell reference signatures rather than their literal presence in blood. Importantly, xCell scores are enrichment scores inferred from marker gene sets and are not direct measurements of cell abundance. With only 16 samples, the correlation estimates—including those in the 0.8–0.9 range—are particularly unstable and should not be over-interpreted. These results therefore provide only weak, hypothesis-generating clues about a possible association between *CLOCK*/*MARK2* and peripheral immune cell states, and they do not establish immune microenvironment remodeling in T2DM.

### 3.4. Scrna-Seq Reveals the Key Role of Monocytes in T2DM

**Figure 4:**
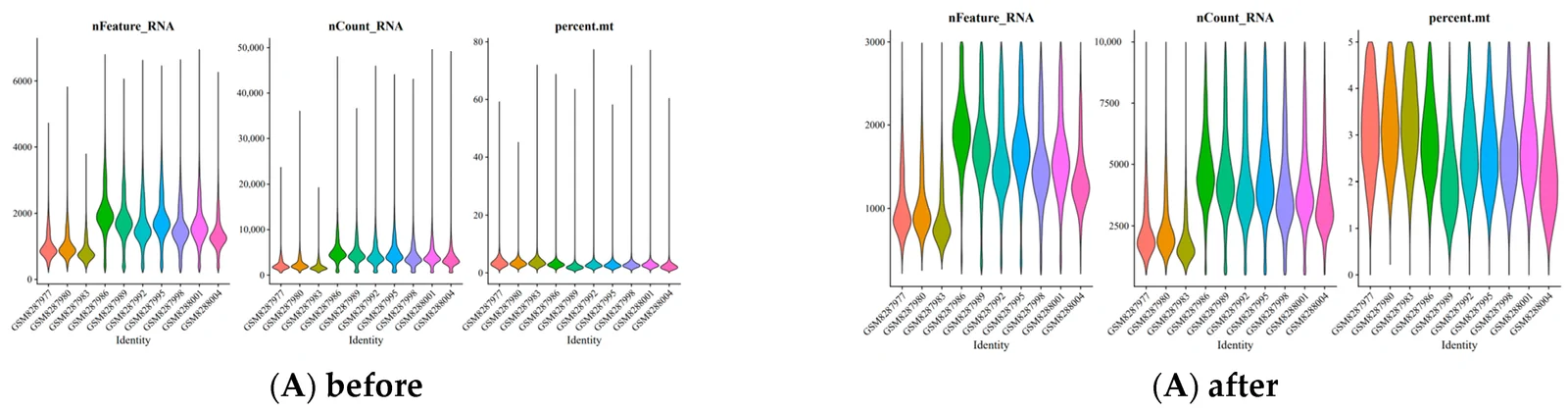

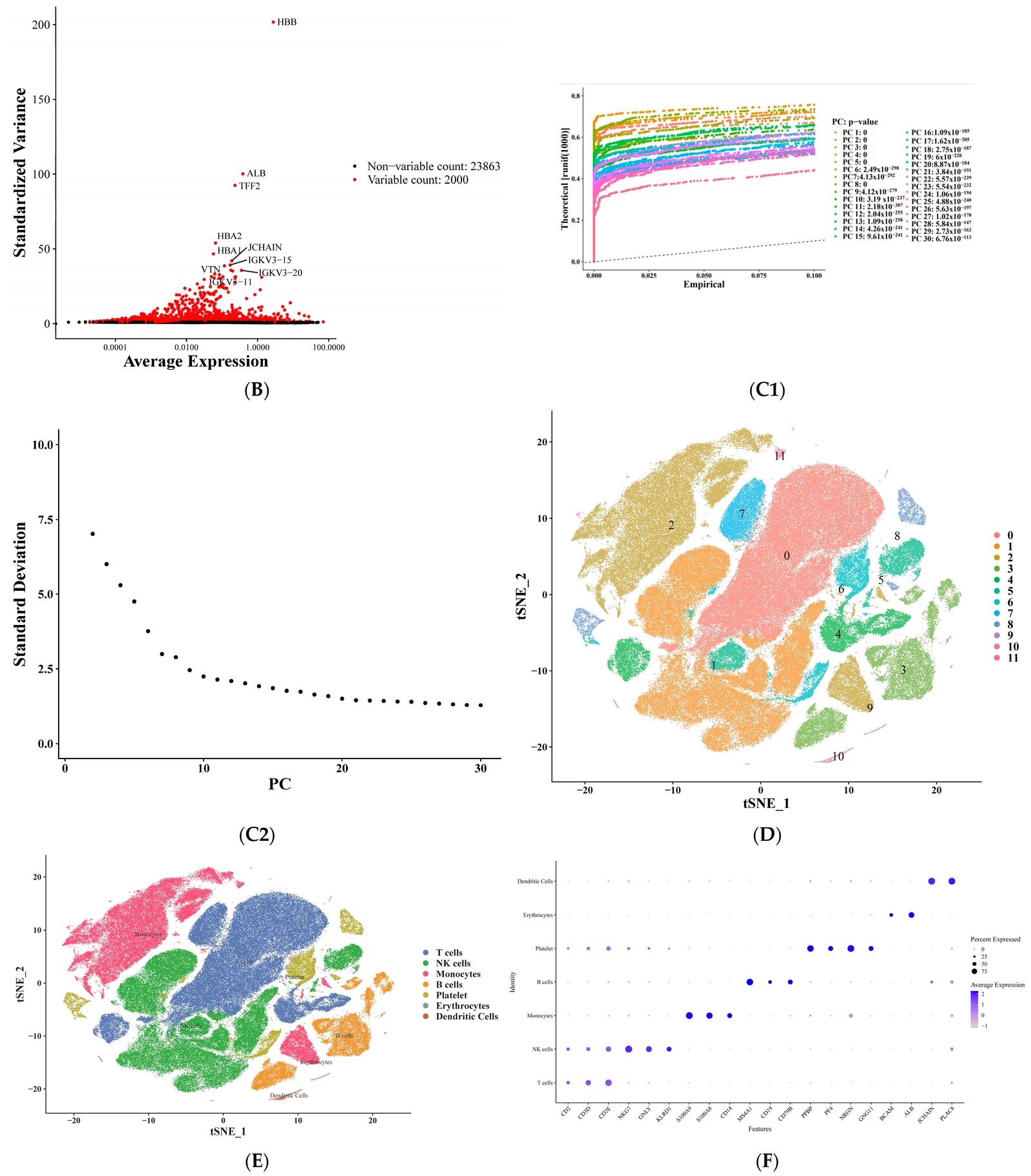

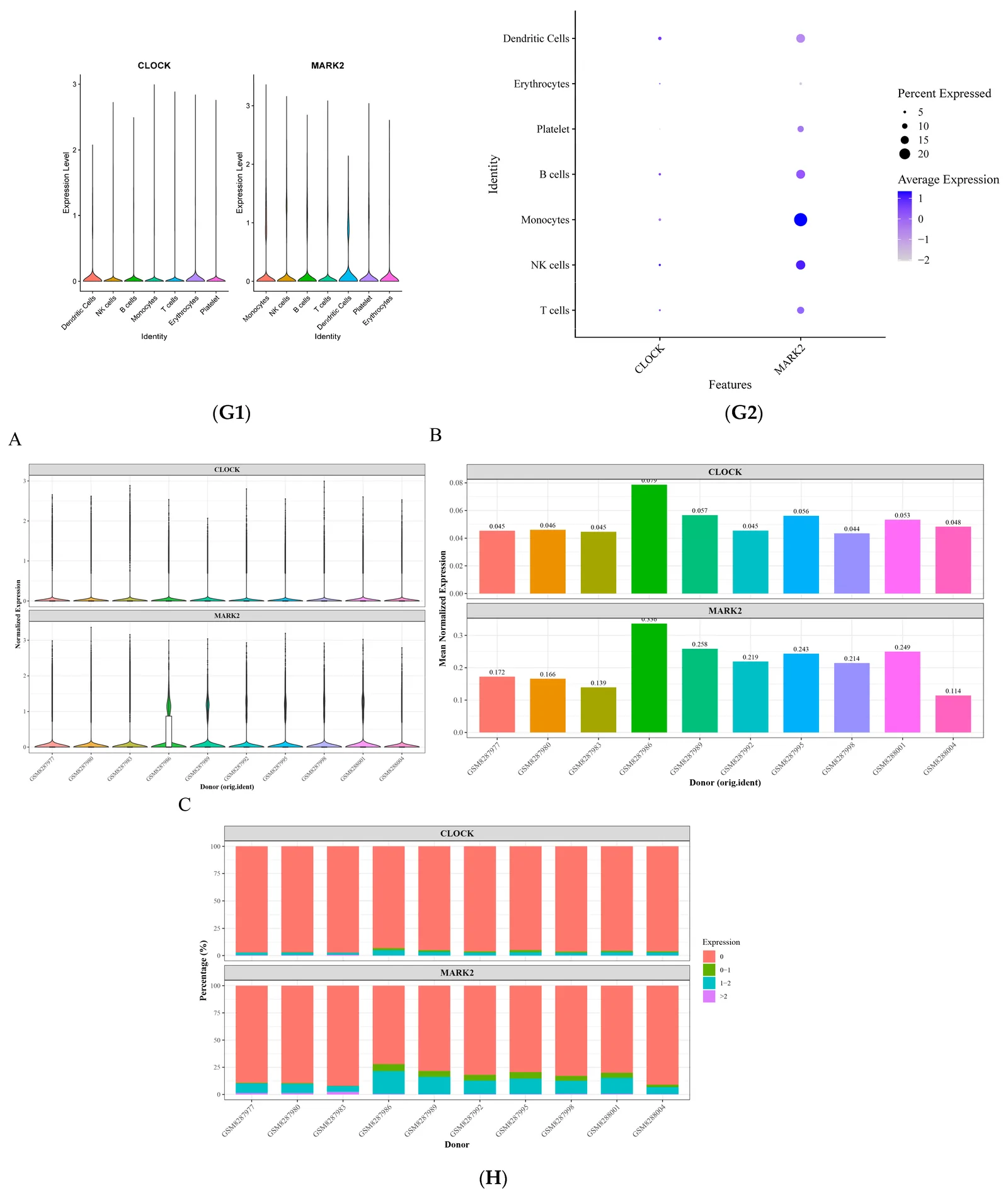
Single-cell RNA sequencing reveals the critical role of monocytes in T2DM. (**A**) Quality control metrics (nCount_RNA, nFeature_RNA, percent.mt) before and after filtering for the GSE268210 dataset (10 T2DM patients). After QC, 209,013 cells remained from an initial 244,560 cells. (**B**) Top 2000 highly variable genes (HVGs) identified by variance stabilization transformation (VST), with the top 10 HVGs labeled. (**C1**,**C2**) Jackstraw permutation test *p*-values and elbow plot for principal component analysis (PCA), showing that the first 20 PCs were retained (*p* < 0.05). (**D**) t-distributed stochastic neighbor embedding (tSNE) plot showing 12 cell clusters at resolution = 0.1. (**E**) Annotated tSNE plot showing seven cell subpopulations: T cells, NK cells, monocytes, B cells, platelets, erythrocytes, and dendritic cells. (**F**) Dot plot showing expression patterns of marker genes used for cell type annotation. (**G1**,**G2**) Expression levels of *CLOCK* and *MARK2* across cell subpopulations, showing highest expression in monocytes. Spearman correlation between *CLOCK* and *MARK2* in monocytes: r = 0.52, *p* < 0.001. (**H**) Donor-stratified expression distributions of *CLOCK* and *MARK2* in GSE268210. For each donor group (**A**–**C**), the five-number summary (min, Q1, median, Q3, max) of normalized expression is shown. This analysis assessed inter-donor variability and the influence of individual donor on overall expression patterns. As the dataset lacked healthy controls, this visualization characterizes inter-individual heterogeneity, not disease-specific expression. Sample size: GSE268210, 10 T2DM donors; no healthy controls were included. After quality control, 209,013 cells were retained. Cluster marker genes were identified using FindAllMarkers (min.pct ≤ 0.25, only.pos = TRUE, logfc.threshold = 1), with Bonferroni-adjusted *p*-values according to the Seurat default. Spearman correlation between *CLOCK* and *MARK2* in monocytes is shown in (**G**). Donor-stratified analysis in (**H**) summarizes inter-individual heterogeneity; because no healthy controls were available, this analysis does not infer disease-specific expression. Abbreviations: HVGs, highly variable genes; VST, variance stabilization transformation; PCA, principal component analysis; t-SNE, t-distributed stochastic neighbor embedding; QC, quality control.

Through single-cell transcriptome sequencing, the cellular landscape of T2DM patients was mapped out. The initial dataset comprised 244,560 cells and 25,863 genes. After rigorous quality control, 209,013 cells remained—along with all 25,863 genes—for downstream analysis (Figure 4A). Following data normalization, we identified 2000 highly variable genes (HVGs) (Figure 4B). Principal component analysis further revealed that the top 20 principal components captured significant biological signals (*p* < 0.05) (Figure 4C1,C2). Unsupervised clustering grouped the cells into 12 distinct clusters at a resolution of 0.1 (Figure 4D). These were then annotated into seven cell subpopulations: T cells, NK cells, monocytes, B cells, platelets, erythrocytes, and dendritic cells (Figure 4E). The expression patterns of specific marker genes for these subpopulations were visualized using bubble plots (Figure 4F). Investigation of key gene expression across cell populations suggested that both *CLOCK* and *MARK2* exhibited the highest expression levels in monocytes compared to other immune cell types (Figure 4G1,G2). To further assess inter-donor variability, *CLOCK* and *MARK2* expression was summarized separately for each of the 10 T2DM donors. *CLOCK* was detectable in all donors, with the donor-level mean normalized expression ranging from 0.0435 to 0.0787 and the proportion of CLOCK-expressing cells ranging from 2.55% to 6.84%. *MARK2* was also detected in all donors, with the mean normalized expression ranging from 0.1140 to 0.3363 and the proportion of *MARK2*-expressing cells ranging from 7.90% to 27.89%. Thus, the overall expression of both genes was represented across multiple donors rather than being attributable to a single sample, although appreciable inter-donor heterogeneity was observed, particularly for MARK2 (Figure 4H). Notably, within monocyte clusters, *CLOCK* and *MARK2* showed coordinated expression patterns (Spearman r = 0.52, *p* < 0.001), suggesting their co-regulation in monocytes. These findings indicate that monocytes represent a major peripheral cellular compartment exhibiting *CLOCK* and *MARK2* expression among the T2DM donors analyzed. Because GSE268210 lacks healthy controls, these observations are strictly descriptive of the T2DM cellular landscape; they do not confirm disease specificity, validate the diagnostic signature, or establish that monocyte enrichment is unique to T2DM. Future studies incorporating healthy donor scRNA-seq data are needed to determine whether these features distinguish T2DM from normal physiology.

### 3.5. Monocyte Differentiation Trajectory and Functional Status in the T2DM Immune Microenvironment

**Figure 5:**
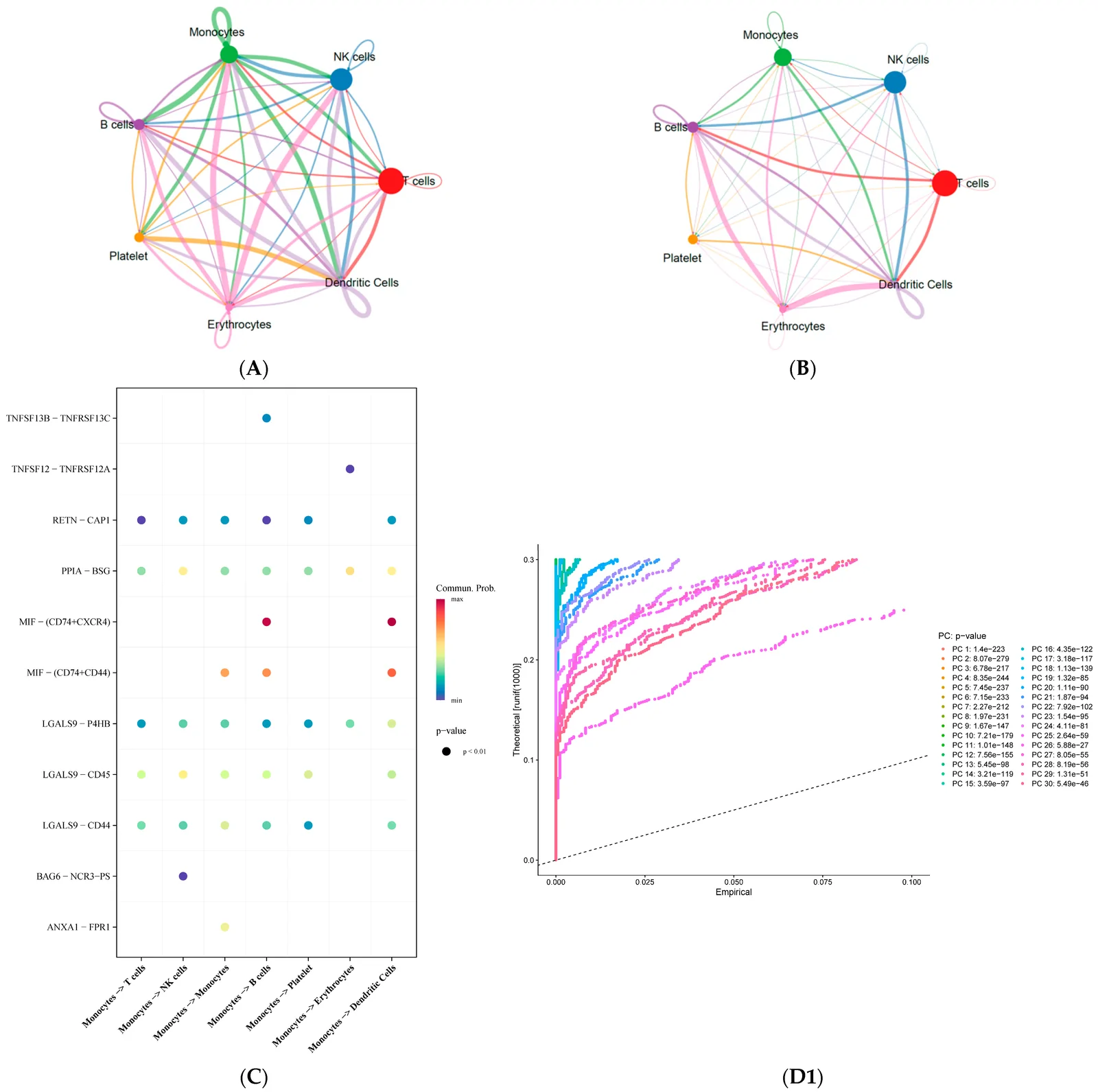

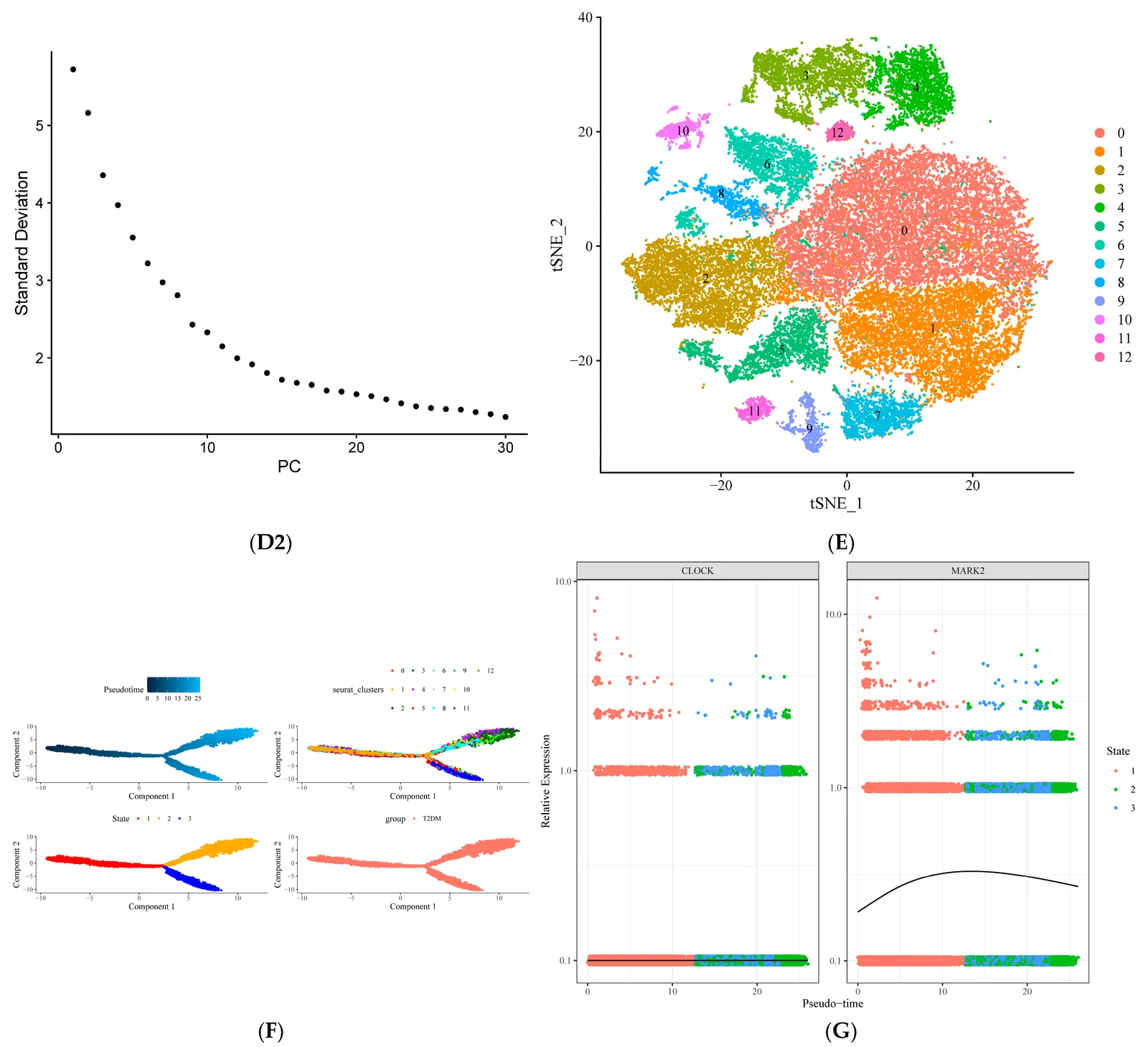

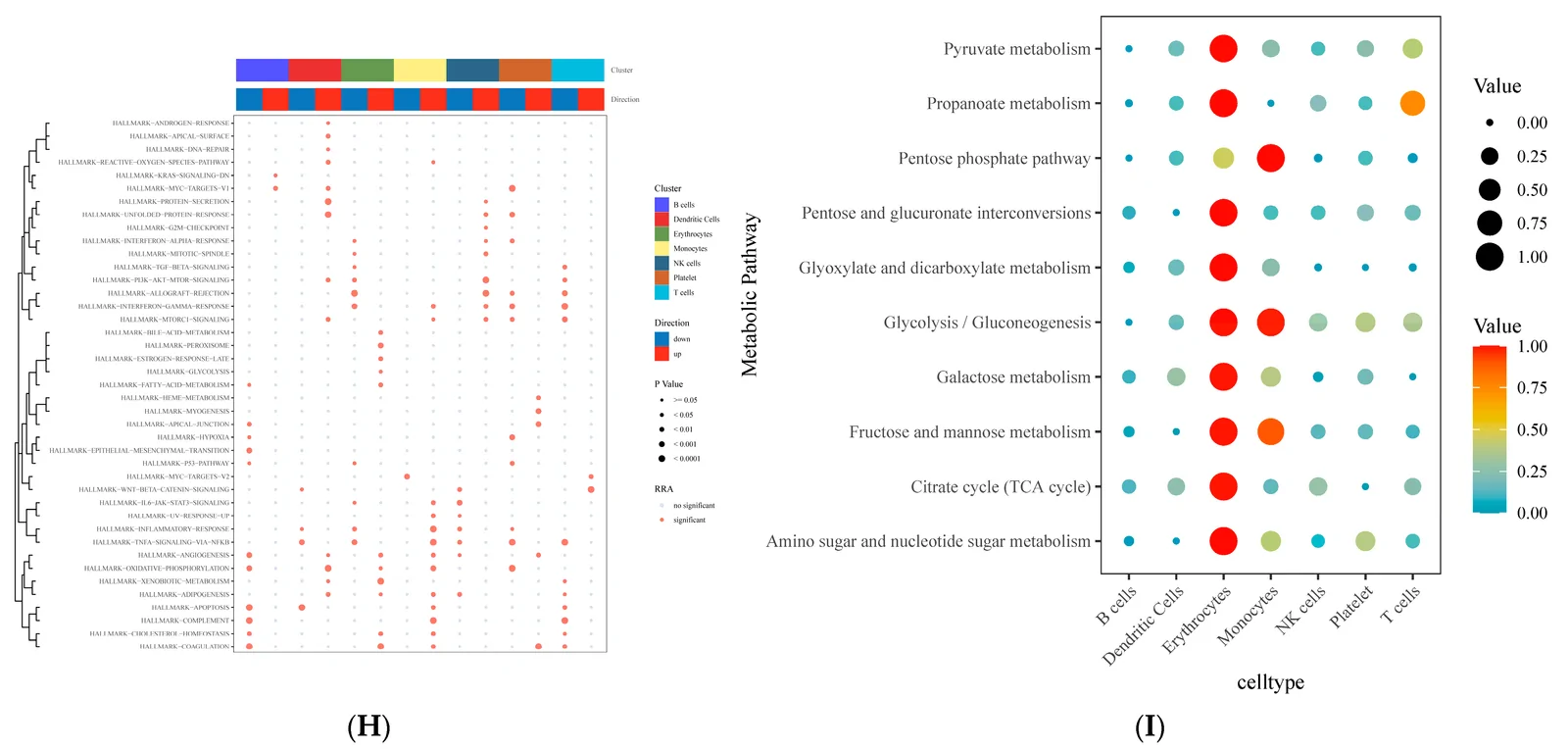
Monocyte differentiation trajectory and functional states in the T2DM immune microenvironment. (**A**–**C**) Cell communication analysis showing monocyte interactions with B cells and dendritic cells, with MIF-(CD74+CXCR4) as the top interaction. (**D1**–**F**) Pseudotime trajectory (13 clusters, 3 states) showing *CLOCK* remaining stable and MARK2 peaking early. (**G**) TNFA-NFκB and oxidative phosphorylation activity across pseudotime. (**H**,**I**) Metabolic pathway activity scores in monocytes (pentose phosphate pathway, glycolysis/gluconeogenesis, and fructose/mannose metabolism). Sample size: GSE268210, 10 T2DM donors; no healthy controls were included. Cell–cell communication in (**A**–**C**) was inferred using CellChat. Pseudotime trajectories in (**D1**–**F**) were constructed using Monocle. Pathway activity scores in (**G**–**I**) were calculated using irGSEA and scMetabolism. Differential pathway activity across monocyte states was assessed using the Wilcoxon rank-sum test; *p*-values are nominal and were not corrected for multiple testing. Abbreviations: MIF, macrophage migration inhibitory factor; TNFA, tumor necrosis factor alpha; NF-κB, nuclear factor kappa B; OXPHOS, oxidative phosphorylation; PPP, pentose phosphate pathway.

Cell communication analysis within the T2DM scRNA-seq dataset revealed that monocytes exhibited a high number of strong interactions with B cells and dendritic cells. The interaction with the strongest communication probability was MIF-(CD74+CXCR4) (Figure 5A–C). To characterize transcriptional state transitions among monocytes within the T2DM-only single-cell dataset, pseudotime analysis was performed. Dimension reduction and clustering of monocytes were first performed, resulting in 13 clusters (dis = 30, resolution = 0.4) (Figure 5D1–E). Pseudotime analysis identified a total of two branches and three differentiation states, where dark blue represents the early stage, and light blue represents the late stage (Figure 5F). The analysis showed that Cluster 1 was mainly enriched in the early stage of differentiation, while Clusters 4, 9, and 12 were localized in the late stage (Figure 5F). Pseudotime trajectory analysis showed that the two key genes followed very different expression patterns as monocytes differentiated. *CLOCK* levels were relatively steady across all differentiation states. *MARK2* levels, in contrast, rose first and then dropped off moderately (Figure 5G). *MARK2* showed relatively higher expression in earlier inferred pseudotime states, whereas *CLOCK* likely acts as a steady regulator supporting baseline monocyte functions. The corresponding monocyte states also exhibited variation in TNFA-NFκB inflammatory signaling and oxidative phosphorylation pathway scores, revealing a transcriptional co-variation pattern among *MARK2* expression, inflammatory signaling, and metabolic states within the T2DM samples. Notably, differentiation states with high *MARK2* expression coincided with enhanced activity of TNFA-NFκB signaling and oxidative phosphorylation pathways (Figure 5H). This co-occurrence suggests a potential association between *MARK2* transcriptional levels and immunometabolic states in T2DM monocytes; however, because these data are observational, they do not establish a causal role for *MARK2* in monocyte reprogramming or confirm these patterns as disease-specific. Furthermore, the pentose phosphate pathway, glycolysis/gluconeogenesis, and fructose and mannose metabolism were among the pathways with high metabolic activity in monocytes (Figure 5I). Collectively, the single-cell analyses identified heterogeneous inferred communication, pseudotime-associated transcriptional patterns, and inflammatory and metabolic pathway states among monocytes within the T2DM cohort.

### 3.6. Validation of Key Gene Expression Levels

**Figure 6:**
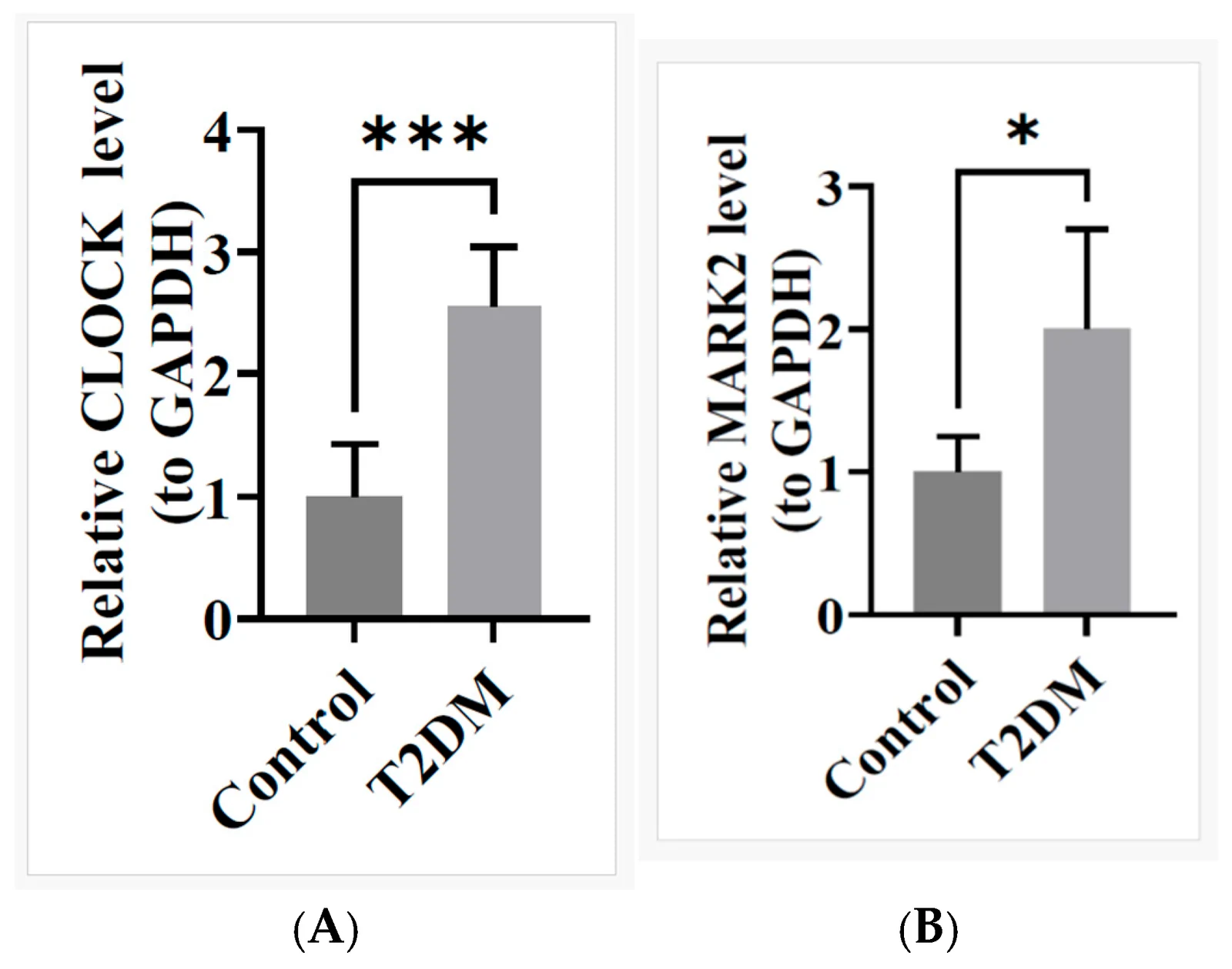
RT-qPCR validation of *CLOCK* and *MARK2*. (**A**) Relative *CLOCK* expression in 5 T2DM patients vs. 5 controls, *CLOCK* was upregulated (*p* = 0.0007; *t*-test). (**B**) Relative *MARK2* expression in 5 T2DM patients vs. 5 controls. *MARK2* was upregulated (*p* = 0.016; *t*-test). Both genes were upregulated, consistent with the bioinformatics results.Sample size: 5 T2DM patients vs. 5 healthy controls. Relative expression was normalized to GAPDH and calculated using the 2^−ΔΔCt^ method. Between-group differences were assessed using an unpaired *t*-test; *p*-values are shown. Given the small sample size (*n* = 5 per group), normality was not formally assessed, and the *t*-test should be regarded as an exploratory test. Error bars represent the mean ± SD. Abbreviations: RT-qPCR, reverse transcription quantitative polymerase chain reaction; GAPDH, glyceraldehyde-3-phosphate dehydrogenase.* *p* < 0.05, *** *p* < 0.001 (*t*-test).

RT-qPCR validation confirmed that both *CLOCK* and *MARK2* were markedly elevated in the T2DM group relative to controls (*p* = 0.0007 and *p* = 0.016, respectively), corroborating the findings from our bioinformatics screening (Figure 6A,B).

Exploratory analyses that are not used to support the primary conclusions are presented in the Supplementary Materials. These include nomogram construction and decision curve analysis (Figure S2), drug prediction (Table S11) and molecular docking (Figure S3 and Table S12), and molecular dynamics simulations (Figure S4).

## 4. Discussion

In this study, we identified *CLOCK* and *MARK2* as T2DM-associated candidate genes through a multi-tiered analytical framework integrating multi-algorithm screening, independent cohort validation, and RT-qPCR confirmation in clinical samples. This design, which combines cross-cohort expression evaluation with preliminary experimental verification, addresses some of the common limitations of bioinformatics studies that rely on a single dataset or lack experimental support. However, given the modest discriminatory performance observed in the larger validation cohort and the descriptive nature of the single-cell findings, these results support *CLOCK* and *MARK2* primarily as T2DM-related candidate biomarkers with consistent cross-cohort expression patterns, rather than as established standalone diagnostic tools.

T2DM is not only a metabolic disease but also a significant risk factor for cognitive impairment and neuropathy. Its prevalence has increased significantly [39]. This context underscores the clinical need for early identification tools for T2DM itself. However, because the present study did not include neurological phenotyping, cognitive assessment, or neuroimaging, the findings should not be interpreted as evidence of neurological involvement; the neural-related gene set served only as a screening starting point.

In this study, *CLOCK* and *MARK2* were identified as candidate key genes through multi-method screening in the GSE15932 training cohort. Both genes consistently showed an upregulated trend in the independent GSE184050 cohort and received further expression support from small-scale clinical RT-qPCR samples. The RT-qPCR should be regarded as preliminary experimental confirmation rather than independent diagnostic evidence, and larger prospectively recruited cohorts with full clinical annotation are required, as described in the Section 6. Exploratory single-cell transcriptomic analysis additionally showed that *CLOCK* and *MARK2* were predominantly expressed in peripheral blood monocytes within the T2DM cohort, where their expression co-occurred with MIF-(CD74+CXCR4)-mediated intercellular communication, TNFA-NFκB inflammatory signaling, and oxidative phosphorylation pathway activation. Due to the lack of healthy controls in the single-cell cohorts (see Section 6), these findings only describe T2DM status. Validation in independent cohorts with healthy donors is required.

The present study was based primarily on peripheral blood transcriptomic data, and detailed individual-level information on age, sex, neurological status, cognitive function, and diabetes-related complications was lacking across the included public cohorts. In particular, no neurological phenotyping, cognitive testing, neuroimaging, or neurodegeneration-related biomarkers were included in this study. Therefore, the present data support an association with T2DM itself, whereas links to neurological manifestations remain speculative and require prospective validation in cohorts with matched neurocognitive data. The inclusion of a gene in a neural-related gene list is therefore not equivalent to evidence that it serves as a biomarker of neurological injury in T2DM.

*CLOCK* and *MARK2* showed high individual discriminatory performance in the GSE15932 discovery cohort, with AUCs of 0.938 and 0.984, respectively. However, their discriminatory performance was attenuated in the larger GSE184050 cohort, where the corresponding AUCs were 0.613 and 0.653. Thus, the present findings do not support uniformly high diagnostic discrimination across datasets. Importantly, the two-gene nomogram was constructed and internally evaluated using GSE15932 and was not externally applied as a fixed combined model to GSE184050. Therefore, the GSE184050 analysis should be interpreted as a cross-cohort evaluation of the individual candidate genes rather than an external validation of the combined diagnostic model. The marked drop in AUC from the discovery cohort (0.938 for *CLOCK* and 0.984 for *MARK2*) to the independent validation cohort (0.613 and 0.653) indicates that these genes should not be regarded as standalone diagnostic biomarkers at this stage. Possible reasons for this include differences in platform, sample composition, disease duration, medication use, and unmeasured clinical confounders. Previous transcriptomic and machine learning studies have identified candidate diagnostic biomarkers for T2DM using bulk or single-cell datasets, although the analytical strategies, sample sources, validation designs, and reported diagnostic performance vary across studies. To facilitate a more direct comparison, representative previous studies and the present study are summarized in Table S13. Rather than indicating superiority over existing models, the present study extends previous transcriptomic biomarker research by integrating multi-algorithm feature selection, independent cross-cohort expression and single-gene ROC evaluation, single-cell localization, and preliminary RT-qPCR validation. In addition, the single-cell analyses provide further cellular and functional context for *CLOCK* and *MARK2*, particularly their enrichment in monocytes and their associations with immunometabolic pathways.

Previous studies have also employed single-cell transcriptomics and machine learning approaches to explore T2DM-related candidate biomarkers and have reported varying degrees of discriminatory ability [40]. Compared with these studies, the present study further integrated expression evaluation in independent bulk transcriptome cohorts, single-cell localization, and preliminary RT-qPCR validation, thereby providing multi-level support for the association between *CLOCK* and *MARK2* and T2DM.

*CLOCK* and *MARK2* have a plausible biological basis as blood-based biomarkers for T2DM.

*CLOCK* is a core circadian clock gene involved in metabolic regulation, and dysregulation of the *CLOCK–BMAL1* axis has been linked to T2DM and other metabolic diseases [41,42]. In neuronal and animal models, hyperglycemia-induced O-GlcNAcylation of BMAL1 has been reported to impair neuronal function [43], and circadian disruption in immune cells can promote the release of inflammatory factors such as TNF-α and IL-6 [44]. *MARK2* has been implicated in proteotoxic stress responses through phosphorylation of eIF2α [45], and abnormal *MARK2* activity has been associated with excessive tau phosphorylation in neurodegenerative diseases [46]. T2DM is a recognized risk factor for Alzheimer’s disease [47], and tau pathology has been reported in the brains of T2DM patients [48]. These mechanisms were established in cellular and animal models or in other disease contexts, and the present study did not assess neurological function, cognitive performance, or neurodegeneration-related biomarkers in any cohort. Whether the peripheral blood upregulation of *CLOCK* and *MARK2* observed here reflects or predicts neurological involvement in T2DM therefore remains an open question. Accordingly, *CLOCK* and *MARK2* should be regarded primarily as candidate blood-based biomarkers associated with T2DM, rather than as neural-related biomarkers of neurological injury.

Our study indicates that *CLOCK* and *MARK2* are co-enriched in insulin signaling and glycolysis/gluconeogenesis pathways, suggesting that they may directly regulate the core metabolic processes of peripheral tissues. These findings provide biological context supporting further evaluation of *CLOCK* and *MARK2* as candidate T2DM-associated biomarkers, whereas their potential link to neurological complications remains hypothesis-generating.

Previous studies have reported abnormalities in the molecular circadian *clock* in peripheral leukocytes of patients with T2DM [49], suggesting that circadian rhythm-related signals in peripheral blood may be associated with T2DM to some extent. In this study, *CLOCK* and *MARK2* were detectable in peripheral blood cells and exhibited relatively consistent expression changes in different datasets. However, based on the ROC results from the independent validation cohort, it is more appropriate to regard them as candidate blood biomarkers associated with T2DM at this stage, rather than to infer that they are superior to or can replace existing clinical diagnostic indicators.

The *CLOCK–MARK2* relationship identified here is primarily based on multi-algorithm screening, co-expression analysis (Spearman r = 0.67, *p* = 0.005), and computational network analysis, and no direct experimental evidence currently links the two genes. *CLOCK* is well established in circadian regulation, whereas the association of MARK2 with the circadian–metabolic–immune axis remains speculative. Possible indirect links, such as circadian modulation of intracellular transport and cytoskeletal organization involving *MARK* family kinases, are hypothesis-generating only. Gene perturbation and co-immunoprecipitation experiments are required to test whether a direct molecular relationship exists.

In T2DM patients, monocytes exhibit the highest *CLOCK*/*MARK2* expression among PBMC subsets, suggesting that they may be the primary peripheral cellular compartment for these genes. Because these descriptive single-cell data lack healthy controls and do not establish causality, these findings suggest that monocytes are a major peripheral cell type expressing *CLOCK* and *MARK2* in the T2DM dataset, but they do not establish a pathogenic role or disease-specific involvement. Rather than being passive participants, monocytes may contribute to T2DM-related immunometabolic dysregulation through a triple mechanism of action. First, monocytes undergo immunometabolic reprogramming toward a pro-inflammatory and metabolically activated state. Our results indicate activation of inflammatory pathways such as TNFA-NFκB and complement in T2DM monocytes, alongside enhanced activity of metabolic pathways including oxidative phosphorylation and glycolysis. This is consistent with other studies, where single-cell sequencing showed that monocytes are expanded and metabolically heterogeneous in T2DM [31]. A persistent hyperglycemic environment may dysregulate monocyte functional phenotypes and disrupt immune homeostasis [50]. Second, monocytes construct and maintain an abnormal immune microenvironment through the macrophage migration inhibitory factor (MIF) signaling axis. We found that monocytes engage in high-intensity communication with B cells and dendritic cells via MIF-(CD74+CXCR4) signaling. MIF is a potent pro-inflammatory cytokine, and the co-expression of its receptor CD74 with CXCR4 is closely associated with cell survival and migration [51]. In T2DM, activated monocytes may continuously activate this pathway in themselves and other immune cells through excessive secretion of MIF, forming an autocrine and paracrine pro-inflammatory loop network that not only sustains chronic low-grade inflammation but may also directly promote insulin resistance. Third, monocytes serve as a bridge between systemic inflammation and target organ damage. Activated monocytes in the circulation are highly prone to recruitment into tissues such as adipose tissue and the kidneys under metabolic stress. Recruited monocytes become macrophages, which secrete pro-inflammatory cytokines; these cytokines produce systemic inflammation, disturb glucose regulation, and affect insulin sensitivity in a way that ultimately fosters the development of T2DM [52]. In our study, MARK2 showed relatively higher expression in earlier inferred pseudotime states, whereas *CLOCK* exhibited a comparatively stable expression pattern along the trajectory. Within this framework, *CLOCK* and *MARK2* may represent candidate molecular correlates of monocyte immunometabolic activation rather than direct mediators of target organ damage.

Collectively, *CLOCK* and *MARK2* may be viewed within a broader immunometabolic framework in T2DM. Monocytes showed the highest expression of both genes among peripheral blood cell types, together with enhanced TNFA-NFkB inflammatory signaling and oxidative phosphorylation. These findings are consistent with the concept that chronic hyperglycemia and low-grade inflammation drive monocyte immunometabolic reprogramming, in which inflammatory signaling and metabolic pathways reinforce each other [31,50]. The MIF-(CD74+CXCR4) axis may further amplify monocyte crosstalk with B cells and dendritic cells, contributing to a self-sustaining inflammatory microenvironment [51,52]. Within this context, *CLOCK* and *MARK2* may serve as candidate molecular nodes linking circadian regulation, cellular stress responses, and monocyte immunometabolism in T2DM, although causal relationships remain to be established.

Computational drug prediction followed by molecular docking and molecular dynamics simulations identified captopril as a compound with putative binding to both *CLOCK* and *MARK2* (Figure S4E,F). These findings are purely in silico, do not establish target selectivity or functional inhibition, and are presented as a hypothesis-generating starting point for future experimental screening.

In summary, the present study links *CLOCK* and *MARK2* to a broader immunometabolic context in T2DM. Rather than treating circadian regulation, monocyte activation, inflammation, oxidative stress, and metabolic dysregulation as separate processes, the findings support a model in which these features are interconnected in the peripheral blood compartment. In this model, *CLOCK* and *MARK2* are candidate molecular nodes whose expression in monocytes coincides with inflammatory and metabolic pathway activity. This integrated view is hypothesis-generating and does not establish causality, but it provides a coherent framework for future functional and clinical studies.

## 5. Conclusions

In this study, *CLOCK* and *MARK2* were identified as candidate blood biomarkers associated with T2DM through multi-method screening, independent dataset evaluation, and RT-qPCR validation, with both genes showing relatively consistent expression changes across different cohorts. Their discriminatory ability in the larger independent validation cohort decreased compared with that in the discovery cohort, suggesting that their standalone diagnostic value is relatively limited. Single-cell analysis further provided cellular-level clues regarding the expression of *CLOCK* and *MARK2* in monocytes and their associated immunometabolic states. As neurological phenotypes were not included in this study, the relevant findings cannot be directly extrapolated to diabetic neurological injury, and the term "neural-related" should be understood only as the origin of the screening gene set. Whether these markers are related to neurological complications of T2DM remains to be determined in dedicated cohorts. The RT-qPCR findings are preliminary and should not be interpreted as independent diagnostic evidence. Functional validation in cellular and preclinical models is essential before any translational relevance can be ascribed.

## 6. Limitations

This study has several limitations. First, the discovery cohort (GSE15932, *n* = 16) and the RT-qPCR cohort (*n* = 10) were small. Although *CLOCK* and *MARK*2 showed consistent expression changes in the independent GSE184050 cohort (*n* = 115), their discriminatory performance declined, and the two-gene nomogram was not externally validated as a fixed model. These findings therefore support *CLOCK* and *MARK2* as T2DM-related candidate genes rather than established diagnostic markers. The RT-qPCR cohort (5 vs. 5) also lacked systematic clinical annotation, so the experimental confirmation should be considered exploratory. In addition, the small discovery cohort (8 vs. 8) may be affected by limited statistical power and overfitting; formal stability selection was not performed, and, although multi-algorithm consensus and cross-cohort consistency provide pragmatic robustness checks, overfitting cannot be fully excluded. Larger, multicenter, prospective cohorts are still needed.

Second, the GEO datasets are retrospective and had missing clinical information, including age, sex, diabetes duration, neurological status, cognitive function, and complications. Subgroup and covariate-adjusted analyses could not be performed. This is particularly important because no neurological phenotyping, cognitive testing, neuroimaging, or neurodegeneration biomarkers were included, and *CLOCK* expression may be affected by blood collection timing. Any link between the identified genes and neurological complications therefore remains hypothesis-generating, and the neural-related gene set should be regarded only as a screening starting point.

Third, the single-cell dataset (GSE268210) included only T2DM patients and no healthy controls; the cellular expression, communication, pathway, and pseudotime results therefore describe T2DM characteristics rather than disease-specific changes.

Fourth, the xCell analysis was based on only 16 samples and marker gene-based deconvolution, with no multiple-testing correction; these estimates are unstable and are not direct measurements of immune cell abundance.

Fifth, the *CLOCK*–*MARK2* relationship was inferred from computational and co-expression analyses rather than direct experimental evidence, and the captopril–*CLOCK/MARK2* interactions are based solely on computational predictions. Functional and experimental validation is required.

Sixth, multiple-testing correction was not applied in several exploratory analyses, including enrichment, immune cell infiltration, and single-cell pathway comparisons; these results are hypothesis-generating only.

Finally, for the RT-qPCR cohort *(n* = 5 per group), normality was not formally assessed, and the *t*-test should be regarded as exploratory; non-parametric methods should be used in larger future cohorts.

Nevertheless, the analytical framework established here—integrating multi-algorithm screening, dual-cohort validation, and experimental confirmation—provides a transferable paradigm for evaluating candidate blood-based biomarkers for T2DM. *CLOCK* and *MARK2* showed reproducible cross-cohort expression differences, but their diagnostic value and potential association with neurological complications require further investigation in dedicated cohorts.

## Figures and Tables

**Table 1:** Summary of cohorts, sample characteristics, and analytical roles in the present study.

Cohort	Data Type/Platform	Sample Source	T2DM, *n*	Controls, *n*	Analytical Role	Age/Sex Information	Neurological or Diabetes-Related Complication Information
GSE15932	Bulk transcriptomic microarray; GPL570	Blood	8	8	Discovery/training cohort; differential expression analysis, machine learning feature selection, PPI network analysis, and nomogram construction	Not consistently available for systematic subgroup or covariate-adjusted analyses	Not available for systematic analysis
GSE184050	Bulk transcriptomic dataset; GPL11154	Blood	50	65	Independent validation cohort; evaluation of the expression patterns and cross-cohort reproducibility of candidate key genes	Not consistently available for systematic subgroup or covariate-adjusted analyses	Not available for systematic analysis
GSE268210	Single-cell RNA sequencing; GPL16791	Peripheral blood/PBMCs	10	0	Exploratory single-cell characterization of cell type-specific gene expression, cell–cell communication, pathway states, and pseudotime-associated patterns	Not consistently available for systematic analysis	Neurological phenotyping and healthy control data were not available
Independent clinical cohort	RT-qPCR	Peripheral venous blood	5	5	Preliminary experimental confirmation of *CLOCK* and MARK2 expression	Not reported in the current manuscript	Neurological phenotyping, cognitive assessments, and diabetes-related complication data were not reported

**Note:** T2DM, type 2 diabetes mellitus; PBMCs, peripheral blood mononuclear cells; PPI, protein–protein interaction; RT-qPCR, reverse transcription quantitative polymerase chain reaction. Detailed individual-level demographic and clinical variables, including age, sex, neurological status, cognitive function, and diabetes-related complications, were not consistently available across the public datasets and therefore could not be systematically incorporated into subgroup or covariate-adjusted analyses.

## Data Availability

The datasets analyzed in this study are publicly available from the Gene Expression Omnibus (GEO) repository. The authors do not own the data. The GSE15932 dataset (platform: GPL570), GSE184050 dataset (platform: GPL11154), and GSE268210 single-cell dataset (platform: GPL16791) can be accessed via the following links: https://www.ncbi.nlm.nih.gov/geo/query/acc.cgi?acc=GSE15932 (accessed on 25 September 2025), https://www.ncbi.nlm.nih.gov/geo/query/acc.cgi?acc=GSE184050 (accessed on 25 September 2025), and https://www.ncbi.nlm.nih.gov/geo/query/acc.cgi?acc=GSE268210 (accessed on 25 September 2025), respectively. The reference gene set (c2.cp.kegg.v7.5.1.symbols.gmt) was retrieved from the Molecular Signatures Database (MSigDB https://www.gsea-msigdb.org/gsea/msigdb (accessed on 25 September 2025), https://www.gsea-msigdb.org/gsea/msigdb). The drug prediction analysis utilized the DSigDB database (https://dsigdb.tanlab.org/DSigDBv1.0/) (accessed on 25 September 2025). Three-dimensional protein structures were obtained from the RCSB PDB database (https://www.rcsb.org/) (accessed on 25 September 2025), and ligand structures were retrieved from the PubChem database (https://pubchem.ncbi.nlm.nih.gov/) (accessed on 25 September 2025). Subcellular localization analysis was performed using DeepLoc-2.0 (https://services.healthtech.dtu.dk/services/DeepLoc-2.0/) (accessed on 26 September 2025), and molecular docking was conducted using CB-Dock2 (https://cadd.labshare.cn/cb-dock2/php/index.php) (accessed on 25 September 2025). The analysis scripts used in this study are publicly available at Zenodo: https://doi.org/10.5281/zenodo.22645104. The repository contains the code required to reproduce the principal computational analyses and figures reported in this study.

## Supplementary Materials

The following supporting information can be downloaded at: https://www.mdpi.com/article/10.3390/genes17101262/s1, Figure S1: Immune microenvironment characteristics in the GSE15932 cohort. Figure S2: Nomogram construction and decision curve analysis. Figure S3: Molecular docking. Figure S4: Molecular dynamics simulations. Table S1: List of 1889 neural-related genes (NRGs). Table S2: Primers used for RT-qPCR. Table S3: Differentially expressed genes (DEGs) in the GSE15932 training cohort. Table S4: Candidate genes (CGs) identified from DEG–NRG intersection. Table S5: GO enrichment results of candidate genes. Table S6: KEGG enrichment results of candidate genes. Table S7: Hub genes identified by three CytoHubba algorithms. Table S8: GSEA results for *CLOCK*. Table S9: GSEA results for *MARK2*. Table S10: Correlation between key genes and differentially enriched immune cells (DEICs). Table S11: Drug–gene interaction network predicted by DSigDB. Table S12: Molecular docking results of captopril with *CLOCK* and *MARK2*. Table S13:Comparison with representative T2DM biomarker studies [40,53,54,55]. Methods S1: Nomogram construction and decision curve analysis. Methods S2: Subcellular and Chromosomal Localization Analysis of Kgs. Methods S3: Immune cell infiltration using xCell. Methods S4: Drug Prediction and Molecular Docking. Methods S5: Molecular dynamics simulation.

## Abbreviations

The following abbreviations are used in this manuscript:

T2DM	type 2 diabetes mellitus
KGs	key genes
ROC	receiver operating characteristic
ScRNA-seq	single-cell RNA sequencing
RT-qPCR	reverse transcription quantitative polymerase chain reaction
AGEs	advanced glycation end products
NFL	neurofilament light chain
CNS	central nervous system
HGs	hub genes
DEGs	differentially expressed genes
CGs	candidate genes
GO	Gene Ontology
KEGG	Kyoto Encyclopedia of Genes and Genomes
BPs	biological processes
MFs	molecular functions
CCs	cellular components
PPI	protein–protein interaction
STRING	Search Tool for the Retrieval of Interacting Genes/Proteins
XFGs	XGBoost feature genes
OOB	out-of-bag
RFGs	random forest feature genes
CKGs	candidate key genes
AUC	area under the curve
GSEA	gene set enrichment analysis
DEICs	differentially expressed immune cells
HVGs	highly variable genes
VST	variance stabilization transformation
PCA	principal component analysis
tSNE	t-distributed stochastic neighbor embedding
