# Supplementary material for: Identification of CLOCK and MARK2 as Candidate Blood-Based Biomarkers in Type 2 Diabetes: An Integrated Bulk and Single-Cell Transcriptomic Study
Source: Genes (Basel). 2026 Oct 11;17(10):1262. doi: 10.3390/genes17101262 (PMC13704593; doi:10.3390/genes17101262)
Supplement: Supplementary file 1 [file genes-17-01262-s001.zip › FigureS1-S4/Figure S1/S1-1.pdf]

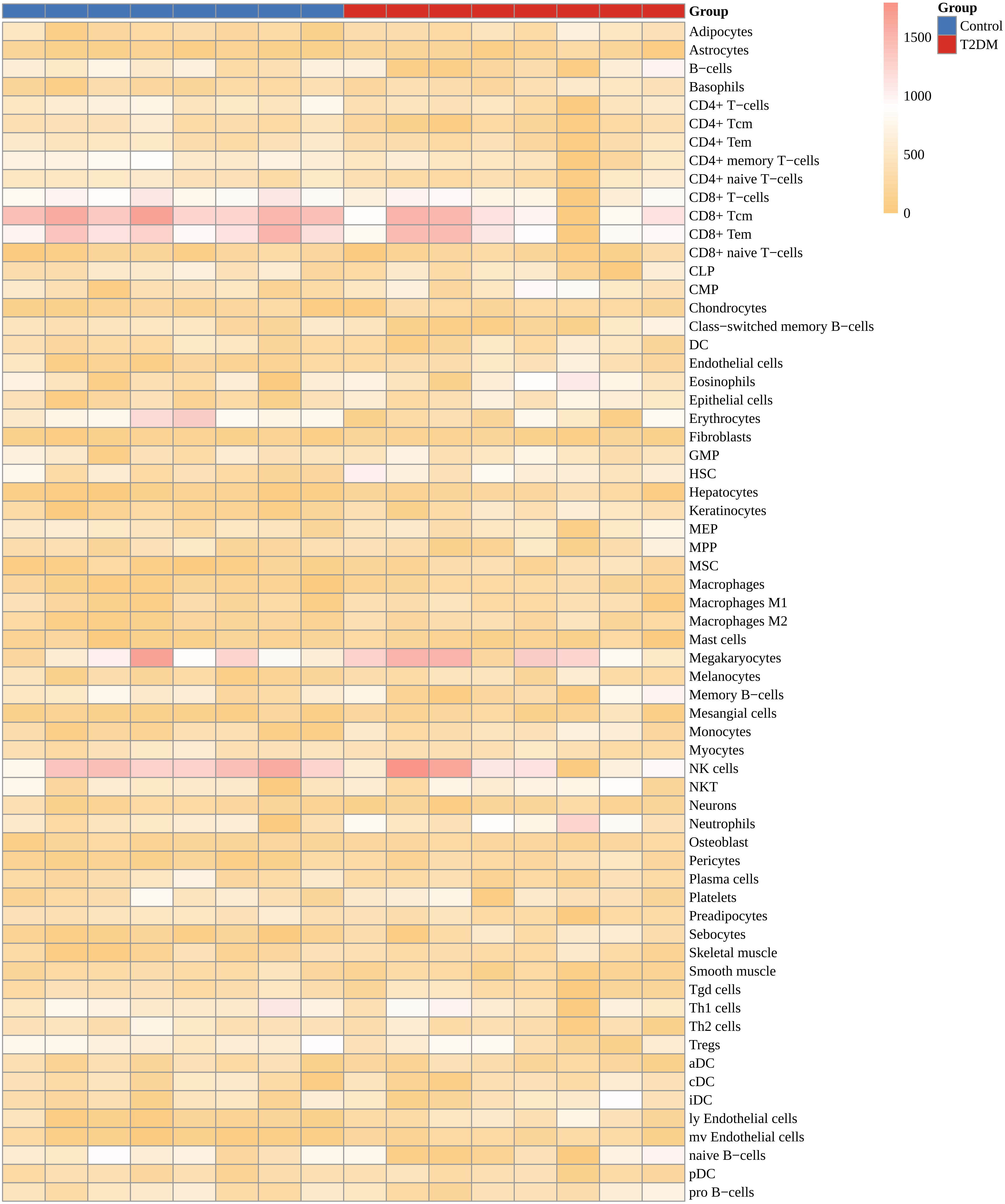
Group
Control
T2DM
1500
1000
500
0
Adipocytes
Astrocytes
B−cells
Basophils
CD4+ T−cells
CD4+ Tcm
CD4+ Tem
CD4+ memory T−cells
CD4+ naive T−cells
CD8+ T−cells
CD8+ Tcm
CD8+ Tem
CD8+ naive T−cells
CLP
CMP
Chondrocytes
Class−switched memory B−cells
DC
Endothelial cells
Eosinophils
Epithelial cells
Erythrocytes
Fibroblasts
GMP
HSC
Hepatocytes
Keratinocytes
MEP
MPP
MSC
Macrophages
Macrophages M1
Macrophages M2
Mast cells
Megakaryocytes
Melanocytes
Memory B−cells
Mesangial cells
Monocytes
Myocytes
NK cells
NKT
Neurons
Neutrophils
Osteoblast
Pericytes
Plasma cells
Platelets
Preadipocytes
Sebocytes
Skeletal muscle
Smooth muscle
Tgd cells
Th1 cells
Th2 cells
Tregs
aDC
cDC
iDC
ly Endothelial cells
mv Endothelial cells
naive B−cells
pDC
pro B−cells
