# Supplementary material for: Identification of CLOCK and MARK2 as Candidate Blood-Based Biomarkers in Type 2 Diabetes: An Integrated Bulk and Single-Cell Transcriptomic Study
Source: Genes (Basel). 2026 Oct 11;17(10):1262. doi: 10.3390/genes17101262 (PMC13704593; doi:10.3390/genes17101262)
Supplement: Supplementary file 1 [file genes-17-01262-s001.zip › FigureS1-S4/Figure S1/S1-2.pdf]

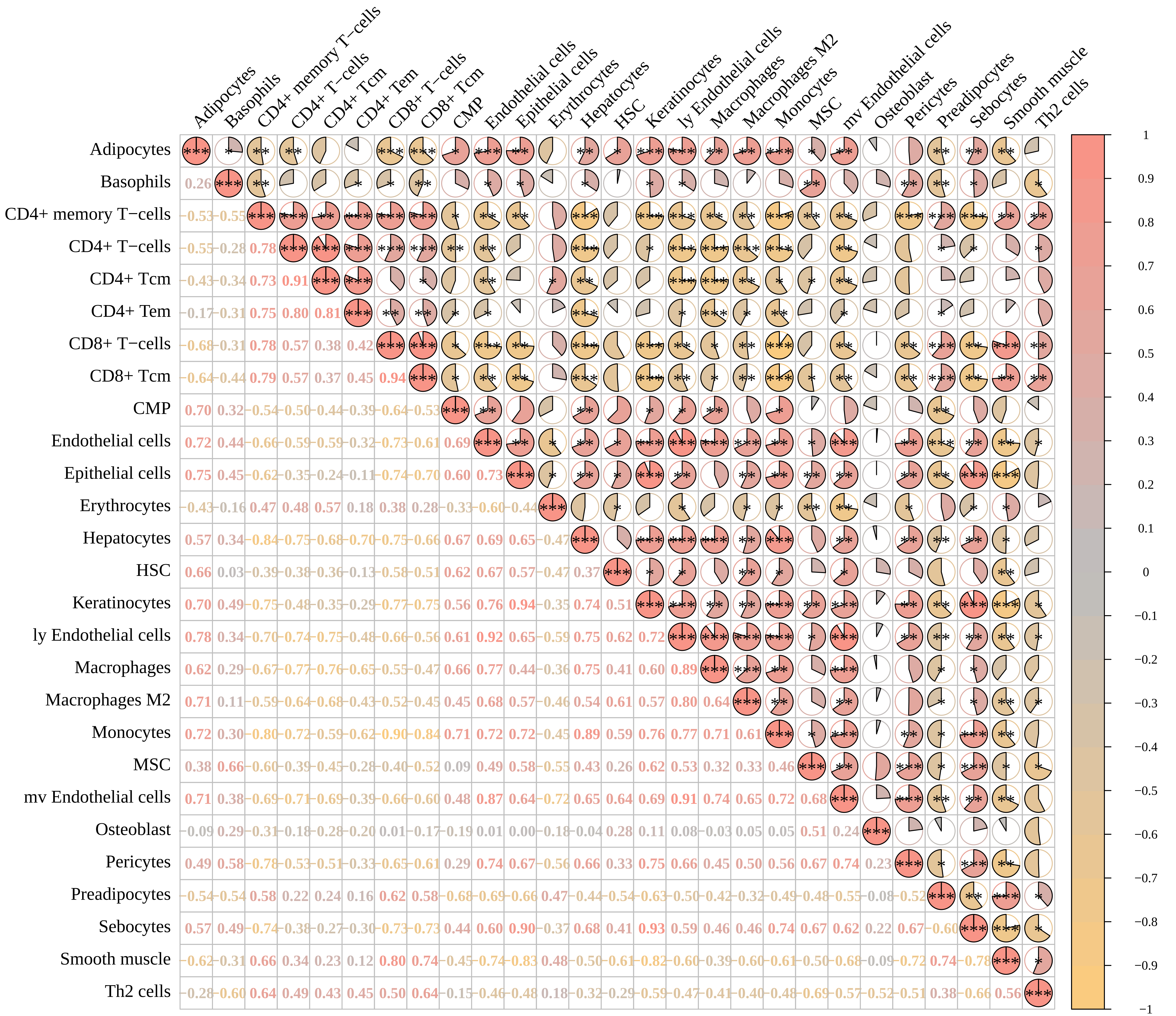
Adipocytes
Basophils
CD4+ memory T−cells
CD4+ T−cells
CD4+ Tcm
CD4+ Tem
CD8+ T−cells
CD8+ Tcm
CMP
Endothelial cells
Epithelial cells
Erythrocytes
Hepatocytes
HSC
Keratinocytes
ly Endothelial cells
Macrophages
Macrophages M2
Monocytes
MSC
mv Endothelial cells
Osteoblast
Pericytes
Preadipocytes
Sebocytes
Smooth muscle
Th2 cells
1
0.9
0.8
0.7
0.6
0.5
0.4
0.3
0.2
0.1
0
−0.1
−0.2
−0.3
−0.4
−0.5
−0.6
−0.7
−0.8
−0.9
−1
