# Supplementary material for: Identification of CLOCK and MARK2 as Candidate Blood-Based Biomarkers in Type 2 Diabetes: An Integrated Bulk and Single-Cell Transcriptomic Study
Source: Genes (Basel). 2026 Oct 11;17(10):1262. doi: 10.3390/genes17101262 (PMC13704593; doi:10.3390/genes17101262)
Supplement: Supplementary file 1 [file genes-17-01262-s001.zip › FigureS1-S4/Figure S2/calibrate.pdf]

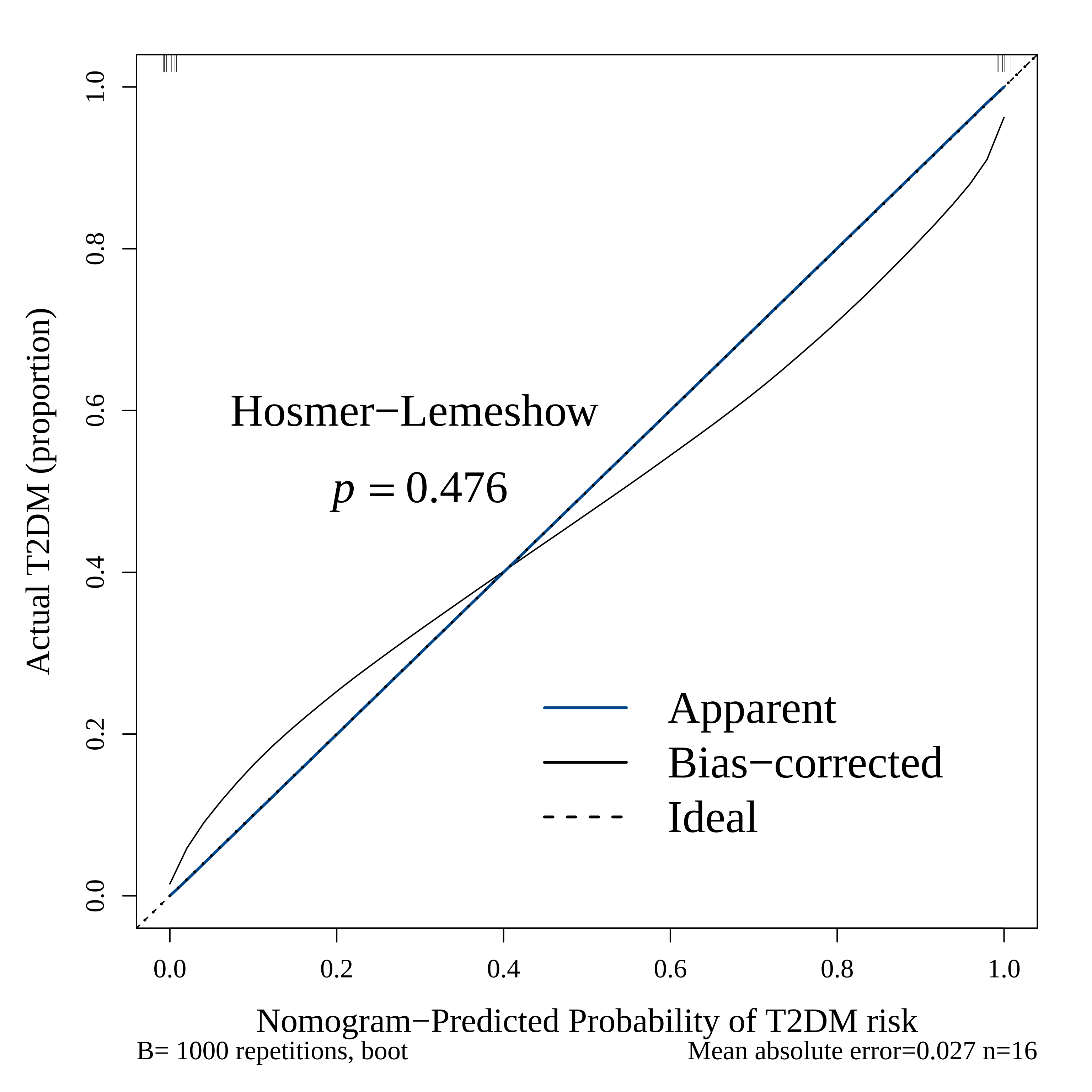

Hosmer−Lemeshow
p = 0.476
Apparent
Bias−corrected
Ideal
Actual T2DM (proportion)
0.0
0.2
0.4
0.6
0.8
1.0
Nomogram−Predicted Probability of T2DM risk
B= 1000 repetitions, boot
Mean absolute error=0.027 n=16
