# Supplementary material for: Identification of CLOCK and MARK2 as Candidate Blood-Based Biomarkers in Type 2 Diabetes: An Integrated Bulk and Single-Cell Transcriptomic Study
Source: Genes (Basel). 2026 Oct 11;17(10):1262. doi: 10.3390/genes17101262 (PMC13704593; doi:10.3390/genes17101262)
Supplement: Supplementary file 1 [file genes-17-01262-s001.zip › FigureS1-S4/Figure S2/Nomogram_line_points_.pdf]

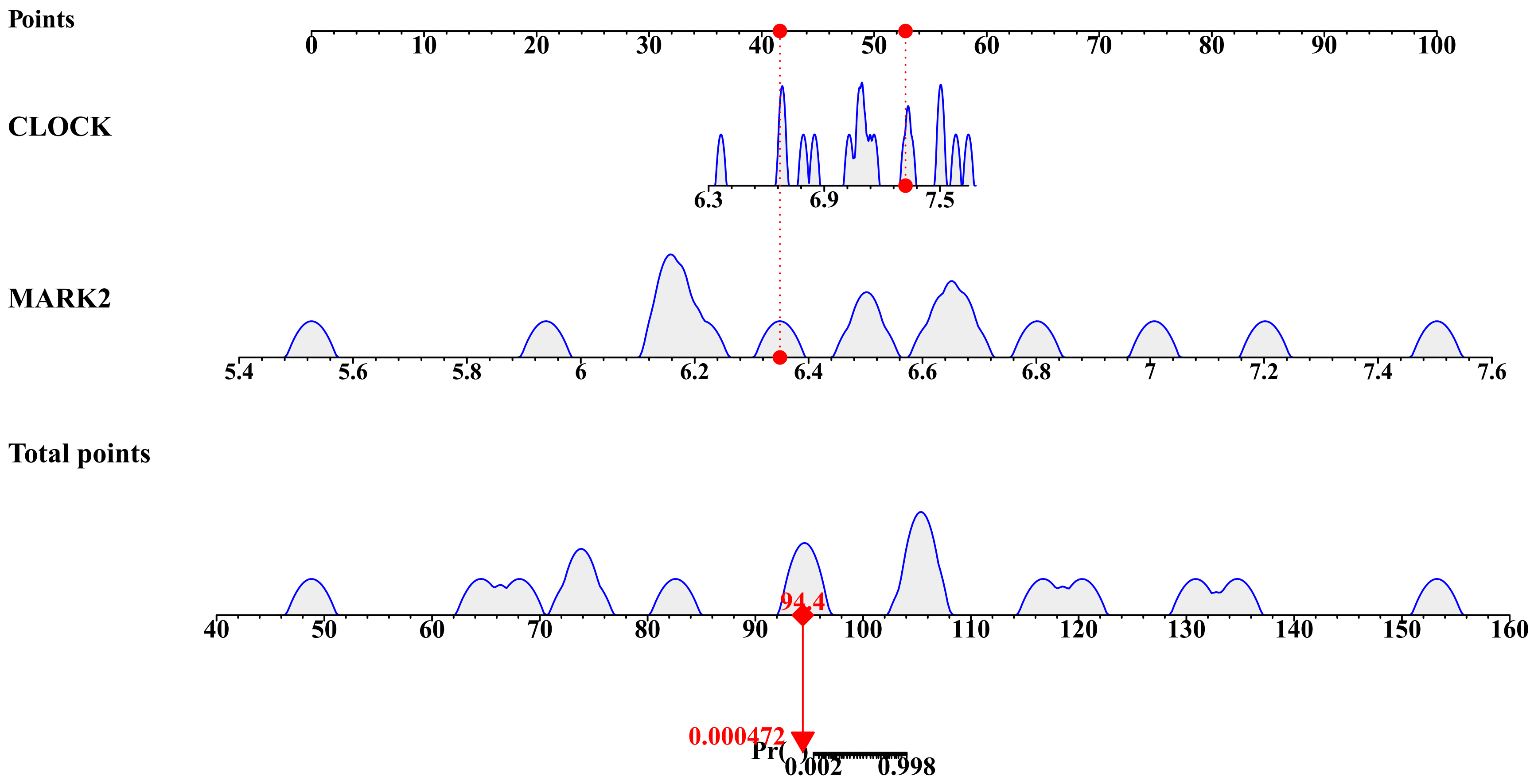

Points
0
10
20
30
40
50
60
70
80
90
100
CLOCK
6.3
6.9
7.5
MARK2
5.4
5.6
5.8
6
6.2
6.4
6.6
6.8
7
7.2
7.4
7.6
Total points
40
50
60
70
80
90
100
110
120
130
140
150
160
94.4
0.000472
Pr( )
0.002
0.998
