# Supplementary material for: Identification of CLOCK and MARK2 as Candidate Blood-Based Biomarkers in Type 2 Diabetes: An Integrated Bulk and Single-Cell Transcriptomic Study
Source: Genes (Basel). 2026 Oct 11;17(10):1262. doi: 10.3390/genes17101262 (PMC13704593; doi:10.3390/genes17101262)
Supplement: Supplementary file 1 [file genes-17-01262-s001.zip › FigureS1-S4/Figure S3/S3A.pdf]

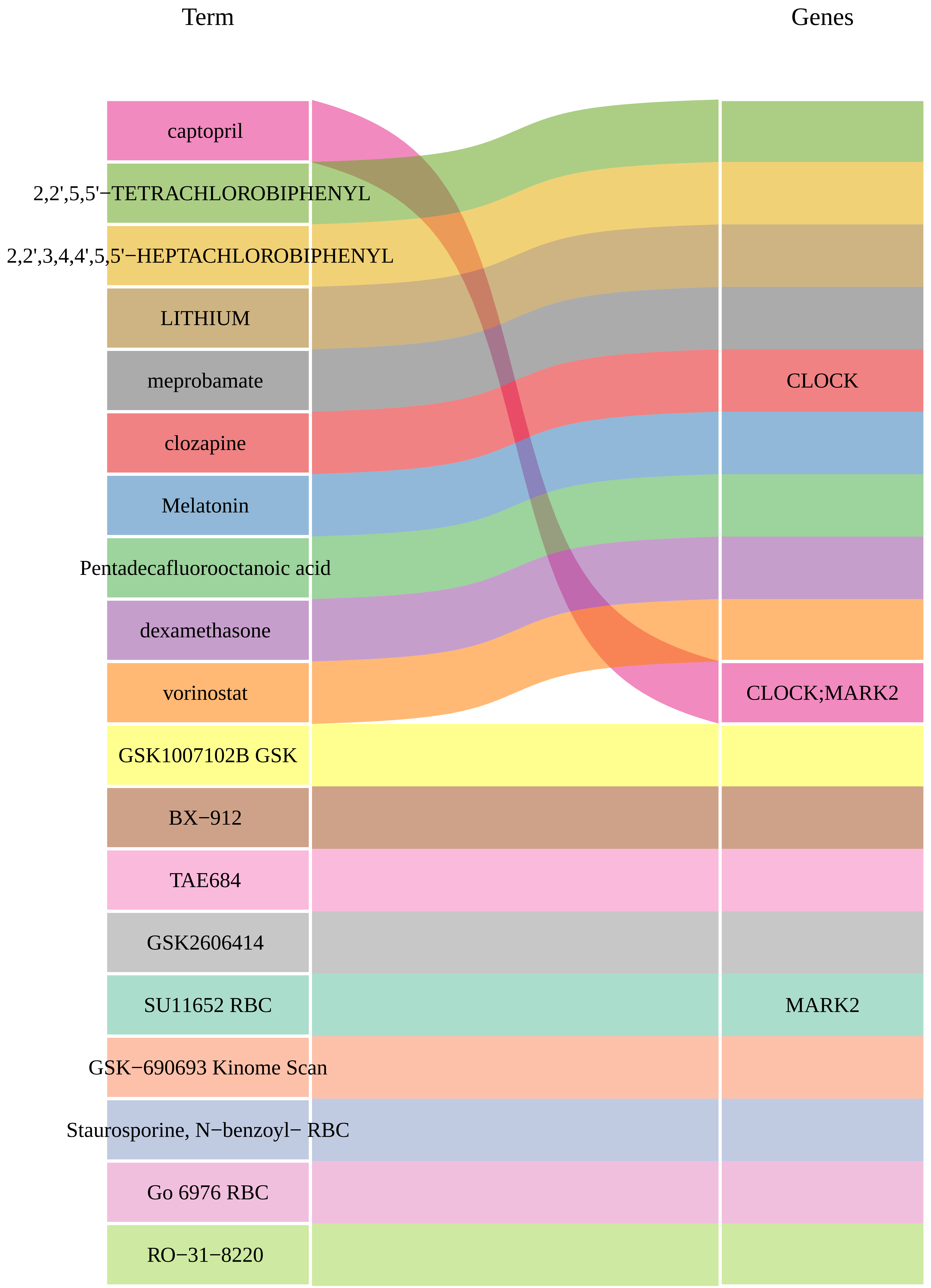

Term
Genes
captopril
2,2',5,5'−TETRACHLOROBIPHENYL
2,2',3,4,4',5,5'−HEPTACHLOROBIPHENYL
LITHIUM
meprobamate
clozapine
Melatonin
Pentadecafluorooctanoic acid
dexamethasone
vorinostat
GSK1007102B GSK
BX−912
TAE684
GSK2606414
SU11652 RBC
GSK−690693 Kinome Scan
Staurosporine, N−benzoyl− RBC
Go 6976 RBC
RO−31−8220
CLOCK
CLOCK;MARK2
MARK2
