# Supplementary material for: Identification of CLOCK and MARK2 as Candidate Blood-Based Biomarkers in Type 2 Diabetes: An Integrated Bulk and Single-Cell Transcriptomic Study
Source: Genes (Basel). 2026 Oct 11;17(10):1262. doi: 10.3390/genes17101262 (PMC13704593; doi:10.3390/genes17101262)

MARK2_captopril

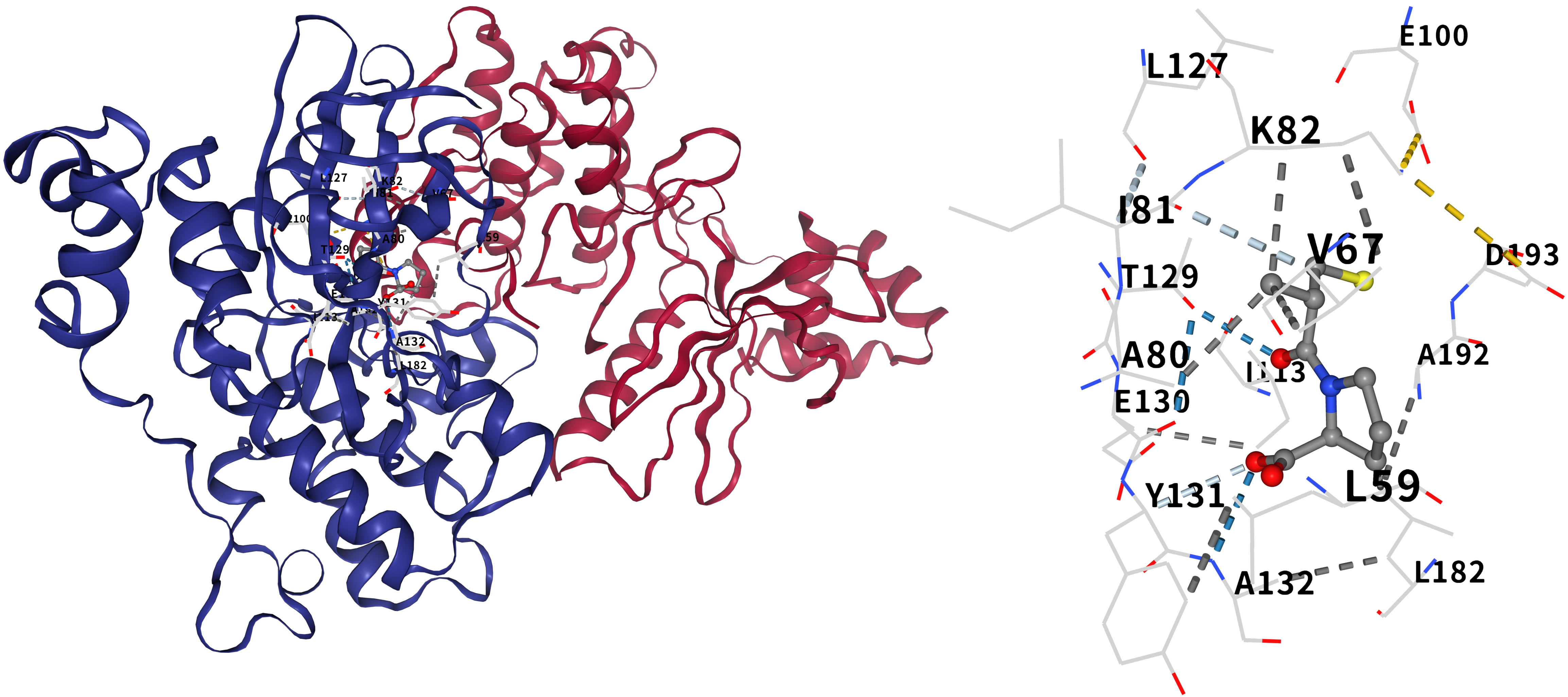

Supplement: Supplementary file 1 [file genes-17-01262-s001.zip › FigureS1-S4/Figure S3/S3C.pdf]
