# Supplementary material for: Identification of CLOCK and MARK2 as Candidate Blood-Based Biomarkers in Type 2 Diabetes: An Integrated Bulk and Single-Cell Transcriptomic Study
Source: Genes (Basel). 2026 Oct 11;17(10):1262. doi: 10.3390/genes17101262 (PMC13704593; doi:10.3390/genes17101262)
Supplement: Supplementary file 1 [file genes-17-01262-s001.zip › FigureS1-S4/Figure S4/Figure S4C.energy_plot.pdf]

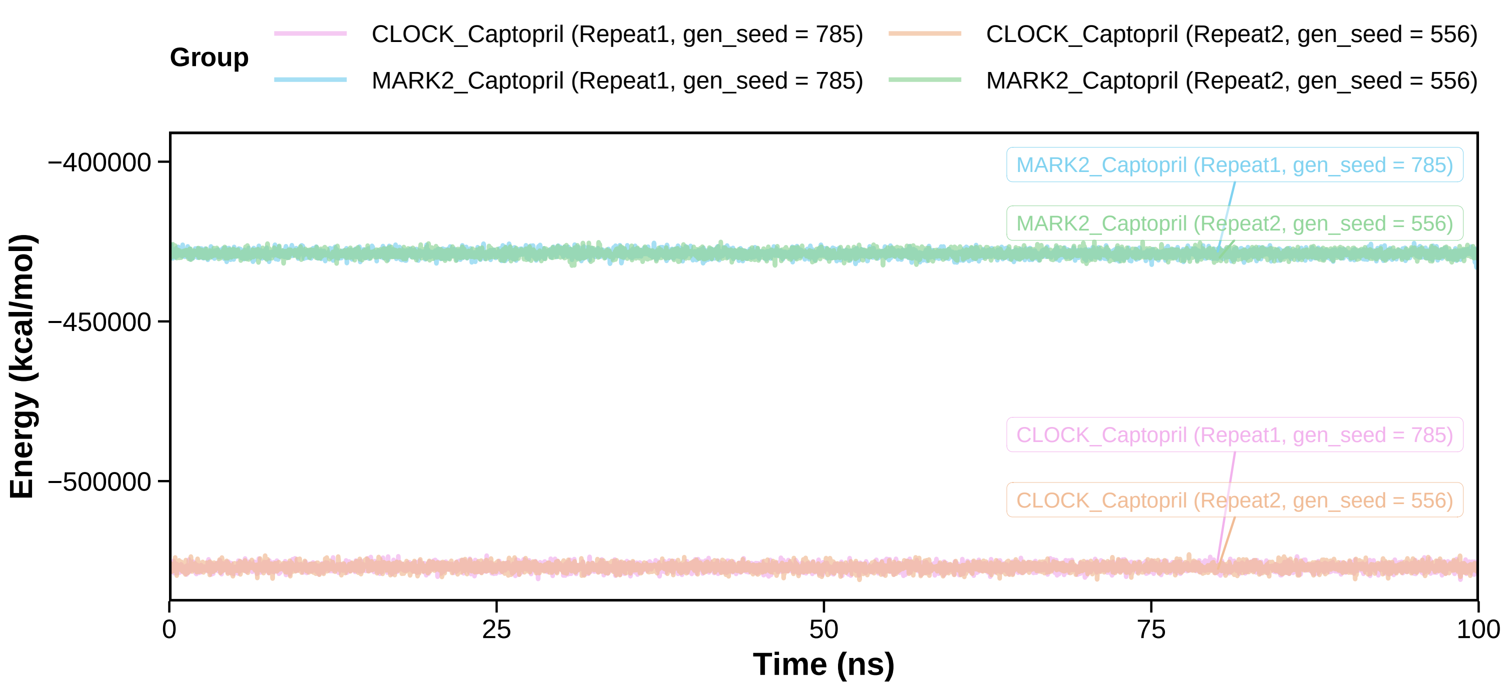

Group
CLOCK_Captopril (Repeat1, gen_seed = 785)
CLOCK_Captopril (Repeat2, gen_seed = 556)
MARK2_Captopril (Repeat1, gen_seed = 785)
MARK2_Captopril (Repeat2, gen_seed = 556)
MARK2_Captopril (Repeat1, gen_seed = 785)
MARK2_Captopril (Repeat2, gen_seed = 556)
CLOCK_Captopril (Repeat1, gen_seed = 785)
CLOCK_Captopril (Repeat2, gen_seed = 556)
−400000
−450000
−500000
Energy (kcal/mol)
0
25
50
75
100
Time (ns)
