# Supplementary material for: Identification of CLOCK and MARK2 as Candidate Blood-Based Biomarkers in Type 2 Diabetes: An Integrated Bulk and Single-Cell Transcriptomic Study
Source: Genes (Basel). 2026 Oct 11;17(10):1262. doi: 10.3390/genes17101262 (PMC13704593; doi:10.3390/genes17101262)
Supplement: Supplementary file 1 [file genes-17-01262-s001.zip › FigureS1-S4/Figure S4/Figure S4E.CLOCK_Captopril_key_dist_plot.pdf]

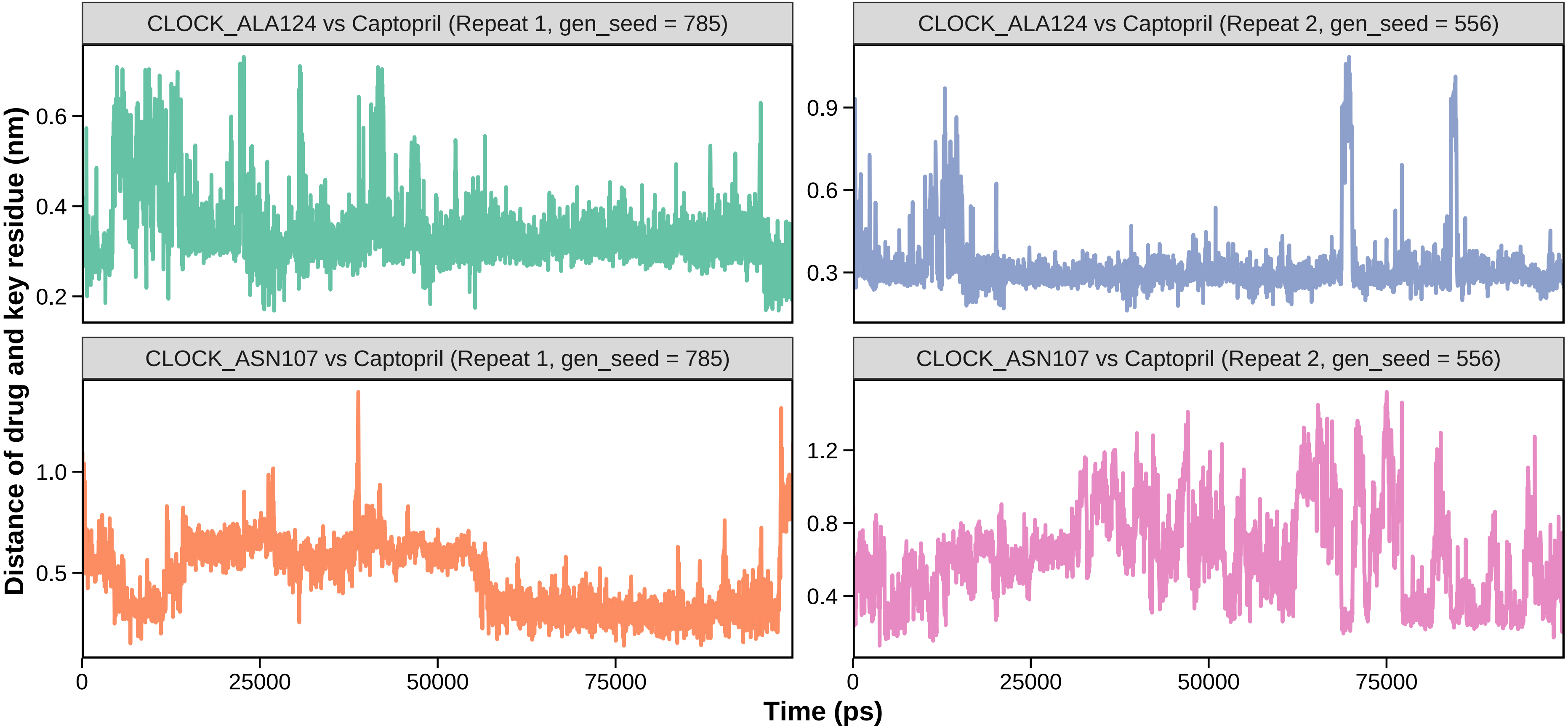
CLOCK_ALA124 vs Captopril (Repeat 1, gen_seed = 785)
CLOCK_ALA124 vs Captopril (Repeat 2, gen_seed = 556)
CLOCK_ASN107 vs Captopril (Repeat 1, gen_seed = 785)
CLOCK_ASN107 vs Captopril (Repeat 2, gen_seed = 556)
Distance of drug and key residue (nm)
0.2
0.4
0.6
0.3
0.6
0.9
0.5
1.0
0.4
0.8
1.2
0
25000
50000
75000
0
25000
50000
75000
Time (ps)
