# Supplementary material for: Identification of CLOCK and MARK2 as Candidate Blood-Based Biomarkers in Type 2 Diabetes: An Integrated Bulk and Single-Cell Transcriptomic Study
Source: Genes (Basel). 2026 Oct 11;17(10):1262. doi: 10.3390/genes17101262 (PMC13704593; doi:10.3390/genes17101262)
Supplement: Supplementary file 1 [file genes-17-01262-s001.zip › Supplementary Methods S1-S5.pdf]

Supplementary Methods S1

nomogram construction and decision curve analysis

To assess the diagnostic potential of the two-gene signature (CLOCK and MARK2), we developed a nomogram using the R package regplot (v 1.1) based on their expression levels in the GSE15932 discovery cohort (8 T2DM vs. 8 controls). In this predictive model, each gene's expression value is mapped to a corresponding score, and these individual scores are summed to generate a total score, where a higher total indicates an increased likelihood of T2DM.

To evaluate the predictive accuracy of the column chart, we used the rms package (version 6.8-1; https://CRAN.R-project.org/package=rms). We assessed model calibration via calibration curve plotting and the Hosmer–Lemeshow goodness-of-fit test, and quantified predictive accuracy using the mean absolute error (MAE) to measure the discrepancy between predicted probabilities and observed outcomes. Decision curve analysis (DCA) was additionally applied to evaluate the model's clinical net benefit across varying threshold probabilities, thereby informing its utility for risk stratification.

Supplementary Methods S2

Subcellular and Chromosomal Localization Analysis of Kgs

To investigate the intracellular locations of the proteins encoded by the KGs, subcellular localization analysis was conducted using the DeepLoc-2.0 online tool (https://services.healthtech.dtu.dk/services/DeepLoc-2.0/). Subsequently, the chromosomal locations of the KGs were visualized using the RCircos package (v 1.2.2).

Supplementary Methods S3

immune cell infiltration using xCell

We estimated the relative abundance of 64 immune cell types in the GSE15932 cohort using the xCell algorithm. Differences in immune infiltration between the T2DM and control groups were evaluated via the Wilcoxon rank-sum test, and cell types with $p < 0.05$ were considered differentially enriched immune cells (DEICs). Spearman correlation analyses were further conducted using the psych package (v 2.2.9) to examine pairwise associations among DEICs, as well as correlations between DEICs and KGs, with statistical significance defined as $|cor| > 0.3$ and $p < 0.05$.

Supplementary Methods S4

Drug Prediction and Molecular Docking

We queried the DSigDB database (https://dsigdb.tanlab.org) to predict candidate compounds targeting the KGs, ranking them by Combined.Score and visualizing the top 10 drug–gene interaction networks. For each KG, we selected the top-ranked compound for further molecular docking to validate the predicted binding interactions. Three-dimensional protein structures of the KGs were downloaded from the RCSB PDB database (https://www.rcsb.org/), and ligand structures were obtained from PubChem (https://pubchem.ncbi.nlm.nih.gov/). Docking simulations were performed using the CB-Dock online platform (https://cadd.labshare.cn/cb-dock2/php/index.php), which calculates binding free energy. Binding affinity was interpreted as follows: |Total Score| > 4.0 kcal/mol indicates a definite binding activity; > 5.0 kcal/mol reflects favorable binding; and > 7.0 kcal/mol denotes potent binding.

Supplementary Methods S5
Molecular dynamics simulation

Following molecular docking, molecular dynamics (MD) simulation was conducted to further validate the stability of the binding conformation between the core drug and the biomarker under more physiologically relevant conditions (explicit water solvent, ions, temperature, and pressure) to assess the stability of the docked complex and to explore its dynamic interaction details.

To further validate the rationality and reliability of the docking results obtained in Section 2.27, we conducted 100 ns molecular dynamics simulations on the protein–ligand complexes using the 'standard dynamics cascade' module of GROMACS (version 2024.2).

A 100 ns simulation was performed for each protein–ligand complex. The AMBER14SB and AMBER gaff force fields were used for the protein and the ligand, respectively. Periodic boundary conditions and the TIP3P water model were applied, and the system charge was neutralized by adding 0.15 mol/L Na^+ and Cl^- ions. Energy minimization was conducted using the steepest descent method, followed by pre-equilibration first under the NVT ensemble (300 K, 100 ps) and then under the NPT ensemble (1 bar, 100 ps).
