# Supplementary material for: Identification of CLOCK and MARK2 as Candidate Blood-Based Biomarkers in Type 2 Diabetes: An Integrated Bulk and Single-Cell Transcriptomic Study
Source: Genes (Basel). 2026 Oct 11;17(10):1262. doi: 10.3390/genes17101262 (PMC13704593; doi:10.3390/genes17101262)
Supplement: Supplementary file 1 [file genes-17-01262-s001.zip › Table S1-S13/Table_S13_Comparison_with_previous_T2DM_biomarker_studies.pdf]

Table S13. Comparison of the present study with representative transcriptomic biomarker studies in type 2 diabetes mellitus

Study	Data / samples	Analytical strategy	Key biomarker(s) / model	Validation and diagnostic performance	Single-cell analysis	Experimental validation	Main contribution
Yang et al., 2023 PMID: 37700362[53]	scRNA-seq: GSE81608 and GSE86469; bulk transcriptome: GSE86468, GSE29226, and external GSE29221	Integrated scRNA-seq and bulk transcriptomics; differential-marker intersection; ROC; pseudotime; functional annotation	CDKN1C and DLK1	Discrimination assessed in GSE86468 and GSE29226 (both AUC > 0.75); external GSE29221 (both AUC > 0.70)	Yes; common cell clusters and pseudotime trajectories were analyzed	Yes; Western blot in primary pancreatic islet cells under high-glucose exposure	Integrated bulk and single-cell evidence to identify T2DM-associated genes and provide cell-state context
Pu et al., 2025 PMID: 39926598[54]	Pancreatic-islet scRNA-seq datasets from T2D and non-diabetic samples	scRNA-seq; metal-ion-transport gene restriction; 12 machine-learning algorithms (108 combinations); ssGSEA; PPI; cell-cell communication	13 hub metal-ion transport-related genes; Stepglm[backward] + GBM model	Training AUC = 0.999; validation AUC = 0.921	Yes; islet cell heterogeneity and cell-cell communication were evaluated	No wet-lab validation reported	Linked a machine-learning signature to metal-ion transport, immune infiltration, and intercellular signaling
Cao et al., 2025 PMID: 40775456[55]	Bulk islet datasets: GSE76895 (training), GSE76894 and GSE86468 (validation); scRNA-seq: E-MTAB-5061	Differential expression; LASSO; SVM-RFE; immune infiltration; GSEA; scRNA-seq localization	SLC2A2	Candidate genes were cross-validated in two independent bulk datasets; SVM-RFE feature-selection model reached AUC = 0.957 in training	Yes; SLC2A2 was localized mainly to beta cells and was reduced in T2D beta cells	Yes; qPCR in a mouse T2D model	Combined multi-cohort bulk validation, single-cell localization, and in vivo experimental confirmation
Tang et al., 2026 PMID: 42205842[40]	scRNA-seq: GSE221156; independent microarray validation: GSE29221; clinical blood cohort: 15 T2DM and 20 controls	scRNA-seq; differential expression; LASSO; ROC; functional enrichment; cell communication	PNLIP, BUB1, CTSB, and NAMPT	Independent-dataset AUCs: 0.819, 0.931, 0.882, and 0.694, respectively	Yes; 10 cell types and disease-associated communication networks were characterized	Yes; qRT-PCR in an independent clinical blood cohort	Integrated single-cell discovery, machine-learning selection, independent validation, and preliminary clinical validation
Present study	Bulk blood datasets: GSE15932 (8 T2DM/8 controls; training) and GSE184050 (50 T2DM/65 controls; validation); scRNA-seq: GSE268210 (10 T2DM); clinical blood cohort: 5 T2DM/5 controls	Neural-related gene restriction; XGBoost; Random Forest; PPI/CytoHubba; ROC; nomogram/DCA; GSEA; xCell; scRNA-seq; CellChat; pseudotime; pathway/metabolic scoring; drug prediction, docking, and MD	CLOCK and MARK2	Both genes showed AUC > 0.90 in the study datasets; expression trends were consistent across training and validation cohorts	Yes; CLOCK and MARK2 were mainly expressed in monocytes; cell communication, pseudotime, functional, and metabolic states were analyzed	Yes; RT-qPCR in an independent clinical blood cohort	Adds a neural-related two-gene blood signature with independent cohort validation, monocyte-level interpretation, experimental confirmation, and exploratory drug-binding analyses

Note: AUC, area under the receiver operating characteristic curve; DCA, decision curve analysis; GBM, gradient boosting machine; MD, molecular dynamics; PPI, protein-protein interaction; qRT-PCR/RT-qPCR, quantitative reverse-transcription polymerase chain reaction; scRNA-seq, single-cell RNA sequencing; T2DM, type 2 diabetes mellitus. Comparisons are descriptive because datasets, tissues, sample sizes, and validation designs differ across studies.

References used for the comparison table:

Yang T, et al. Eur J Med Res. 2023;28:340. doi:10.1186/s40001-023-01321-1. PMID: 37700362.
Pu Z, et al. Front Immunol. 2025;15:1479166. doi:10.3389/fimmu.2024.1479166. PMID: 39926598.
Cao Y, et al. Sci Rep. 2025;15:28924. doi:10.1038/s41598-025-14915-y. PMID: 40775456.
Tang F, et al. Front Med (Lausanne). 2026;13:1801737. doi:10.3389/fmed.2026.1801737. PMID: 42205842.
